# Understanding the development of oral epithelial organs through single cell transcriptomic analysis

**DOI:** 10.1242/dev.200539

**Published:** 2022-08-17

**Authors:** Qianlin Ye, Arshia Bhojwani, Jimmy K. Hu

**Affiliations:** 1 School of Dentistry, University of California Los Angeles, Los Angeles, CA 90095, USA; 2 Molecular Biology Institute, University of California Los Angeles, Los Angeles, CA 90095, USA

**Keywords:** Mandibular development, Ectodermal organs, Patterning, Cell atlas, Gene regulatory networks, Single cell RNA-sequencing

## Abstract

During craniofacial development, the oral epithelium begins as a morphologically homogeneous tissue that gives rise to locally complex structures, including the teeth, salivary glands and taste buds. How the epithelium is initially patterned and specified to generate diverse cell types remains largely unknown. To elucidate the genetic programs that direct the formation of distinct oral epithelial populations, we mapped the transcriptional landscape of embryonic day 12 mouse mandibular epithelia at single cell resolution. Our analysis identified key transcription factors and gene regulatory networks that define different epithelial cell types. By examining the spatiotemporal patterning process along the oral-aboral axis, our results propose a model in which the dental field is progressively confined to its position by the formation of the aboral epithelium anteriorly and the non-dental oral epithelium posteriorly. Using our data, we also identified *Ntrk2* as a proliferation driver in the forming incisor, contributing to its invagination. Together, our results provide a detailed transcriptional atlas of the embryonic mandibular epithelium, and unveil new genetic markers and regulators that are present during the specification of various oral epithelial structures.

## Abstract (precis)

**Summary:** Single cell analysis of embryonic mouse mandibular ectoderm identifies distinct populations with defined gene regulatory networks and unveils epithelial zones that form progressively during oral-aboral patterning.

## INTRODUCTION

The vertebrate mouth is a highly derived structure that consists of specialized organs – teeth, taste buds and glands – that are crucial for feeding and the survival of animals. During development, these ectodermal organs emerge as epithelial thickenings or placodes at precise locations on the oral surface, and the mouse mandible has served as one of the foundational model systems for studying ectodermal organ development (Tucker and Sharpe, 2004; Kapsimali and Barlow, 2013; Suzuki et al., 2021). The mouse lower jaw is formed from the mandibular process of the first pharyngeal arch, which comprises a mesenchymal core ensheathed by a contiguous epithelial layer of ectodermal and endodermal origins (Frisdal and Trainor, 2014). The ectoderm covers the outer mandible and most of the oral surface, whereas the endoderm overlays the inner mandible that extends posteriorly from the proximal part of the tongue to the embryonic foregut (Rothova et al., 2012).

Patterning of the mandibular epithelium is already evident at embryonic day (E) 9.5, when *Bmp4* and *Fgf8* are expressed in the medial (distal) and lateral (proximal) regions of the oral epithelium, respectively, and pattern the incisor and molar fields along the proximal-distal axis (Neubüser et al., 1997; Tucker et al., 1998; Liu et al., 2005). Concurrently, *Shh* is expressed in the pharyngeal endoderm, and signaling by SHH and BMP4 patterns the mandible along the oral-aboral axis (Xu et al., 2019). The mandible is thus organized into broadly patterned domains before the formation of epithelial placodes.

The mandibular arch epithelium begins as a single layer of cuboidal cells but soon undergoes stratification after E9.5 to produce a sheet of flattened cells apically, called the periderm that protects the epithelia from inappropriate fusion (Richardson et al., 2014). Further stratification in the forming ectodermal placodes generates suprabasal cells that stack between the columnar basal layer and the periderm. In the case of the tooth, this thickened epithelium, known as the dental lamina, is discernible at E11.0 and represents the earliest morphological sign of odontogenesis (MacKenzie et al., 1992). However, the exact mechanism that determines the position of the dental lamina and initiates tooth development remains unclear. Cells in the dental lamina are transcriptionally distinct from the rest of the epithelium, expressing several tooth-specific transcription factor genes, including *Pitx2* and *Irx1* (Mucchielli et al., 1997; Yu et al., 2017). In particular, *Pitx2* is crucial for tooth development and is one of the earliest dental markers (Liu et al., 2003). Its expression precedes the lamina stage and is present in a broader domain at E10.5 that later narrows (St Amand et al., 2000). Patterning of the dental field is therefore a progressive process involving dynamic transcriptional changes.

As the dental lamina transitions through the placode and bud stages between E11.0 and E12.5, the initiation knot forms along the anterior tooth epithelium and functions as a signaling center to promote early tooth development (Dassule and McMahon, 1998; Ahtiainen et al., 2016). By E12.0, individual incisor and molar buds are easily recognizable; separated by a toothless space called the diastema, as mouse dentition is reduced and lacks canines and pre-molars. Besides teeth, other oral ectodermal organs also begin to form around this stage. For example, the development of the submandibular salivary gland initiates at E11.5, first as an epithelial thickening adjacent to the tongue, which then protrudes into the mesenchyme as a teardrop shaped bud by E12.0 (Jaskoll and Melnick, 1999). Concomitantly, taste bud primordia develop on the mouse tongue, starting at E12.0 (Mistretta and Liu, 2006). Therefore, E12.0 represents a crucial developmental window for experimental investigations, as it is marked by the emergence and expansion of different progenitor lineages that will form all the major oral ectodermal organs. Crucially, the mechanisms by which diverse mandibular epithelial populations are patterned and specified remain an important unresolved issue. Because studies to date have primarily focused on individual organs or other timepoints (Landin et al., 2012; Musselmann et al., 2011; Hauser et al., 2020; Yuan et al., 2020; Xu et al., 2019), there is incomplete knowledge of the overall cell heterogeneity within the E12.0 mandibular epithelium. It is also not well understood how specific gene regulatory networks and signaling processes are coordinated across both space and time to control the development and functions of different epithelial populations.

To address these questions, we first acquired an in-depth understanding of the cell diversity in the mandibular epithelium at E12.0 using single cell RNA-sequencing (scRNA-seq) (Klein et al., 2015; Macosko et al., 2015). By complementing sequencing results with detailed spatial mapping, we have identified discrete populations not only in the developing ectodermal organs but also in areas where the epithelium appears morphologically simple and uniform. Through computational analysis, we uncovered key transcription factors and associated gene regulatory networks that define these epithelial regions. In addition, we show that the oral-aboral patterning of the mandibular epithelium evolves over time as it becomes increasingly regionalized at the transcriptional level from E8.5 to E12.0. Our findings indicate that the mandibular ectoderm first gives rise to cells expressing tooth-specific transcription factors but subsequent patterning imparts anterior aboral and posterior oral identities to these cells, confining the dental field to its eventual position. Last, we show that *Ntrk2*, a novel dental marker identified in our analysis, promotes cell proliferation and tooth invagination. Our results thus provide a useful resource for future investigation of key regulators during mandibular epithelial morphogenesis.

## RESULTS

### scRNA-seq identifies spatially distinct epithelial populations in the developing mandible

**Fig. 1.**
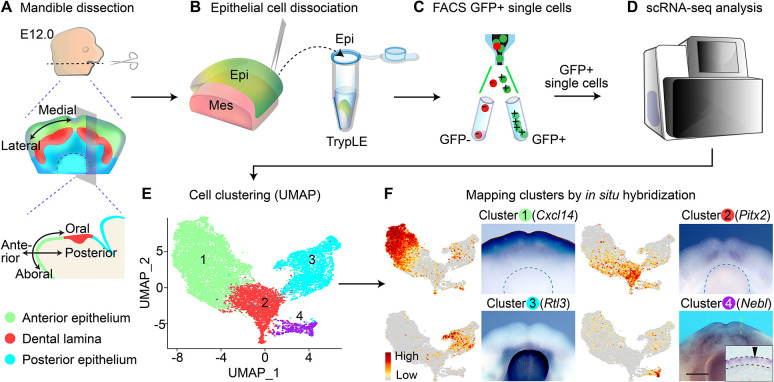
**Single cell RNA-sequencing of E12.0 mandibular epithelium.** (A-D) Workflow of cell isolation from E12.0 mandibular epithelia for scRNA-seq. Schematic drawings of the embryonic mouse mandible in dorsal view and sagittal section showing the anatomical axes and broadly defined epithelial regions. (E) UMAP plot of mandibular epithelial cells. (F) Feature plots of representative markers enriched in the four clusters shown in E and their expression by RNA *in situ* hybridization in E12.0 mandibles (dorsal views). Clusters 1-3 correspond to the anterior (green), dental (red) and posterior (cyan) epithelium, respectively. Blue dashed lines outline the tongue. Cluster 4 (purple) contains periderm cells (black arrowhead). Inset is a representative sagittal section through the region indicated by the yellow dashed line. Black dashed lines indicate the epithelium. Epi, epithelium; Mes, mesenchyme. Scale bar: 400 μm in whole-mount images; 20 μm in the inset.

In order to define the different epithelial populations in the developing mandible based on their genetic differences, we performed scRNA-seq analysis using mandibular epithelia from E12.0 embryos (Fig. 1A-D). At this timepoint, the epithelium has been broadly patterned along different axes (Fig. 1A) (Xu et al., 2019), and oral epithelial organs have just begun to develop and undergo stratification and invagination. The E12.0 mandible therefore provides an ideal platform for investigating the intrinsic genetic regulation governing the development and functions of all the early epithelial progenitor populations that constitute each of the mandibular ectodermal structures and their adjoining regions. Visualized using UMAP, the initial feature plot contains several matching clusters segregated by the expression of cell cycle genes (Fig. S1), reflecting the proliferative nature of a developing tissue. To focus on the transcriptional differences associated with epithelial sub-structures, we subsequently regressed out transcripts related to cell cycles as well as transcripts segregated by sexes.

We first analyzed this refined dataset at a low resolution, and this partitioned the mandibular epithelium into four large clusters (Fig. 1E). Cluster 1 differentially expresses several markers, including *Cxcl14* and *Tfap2b*. To locate these cells in the E12.0 mandible, we performed whole-mount RNA *in situ* hybridization and mapped them to the aboral epithelium that is anterior to the dental lamina and continues to the ventral mandible (Fig. 1F, Fig. S2A). Cluster 2 expresses many known dental markers, such as *Pitx2* (Yu et al., 2020) and *Irx2* (Houweling et al., 2001) (Fig. 1F, Fig. S2B), and thus contains cells from the developing teeth. Analysis of cluster 3 unveiled many previously unidentified markers of the oral epithelium, including *Rtl3* and *Col14a1* (Fig. 1F, Fig. S2C), which are expressed in the epithelium posterior to the forming teeth. The distribution of clusters 1-3 on the UMAP therefore corresponds spatially to the mandibular epithelium along the oral-aboral axis (Fig. 1A,E). Cluster 4 expresses several periderm markers, including *Grhl3* and *Irf6* (Peyrard-Janvid et al., 2014). Detection of other cluster 4 markers, *Nebl* and *Pkp1*, at the epithelial surface confirmed the inclusion of periderm in this cluster (Fig. 1F, Fig. S2D).

**Fig. 2.**
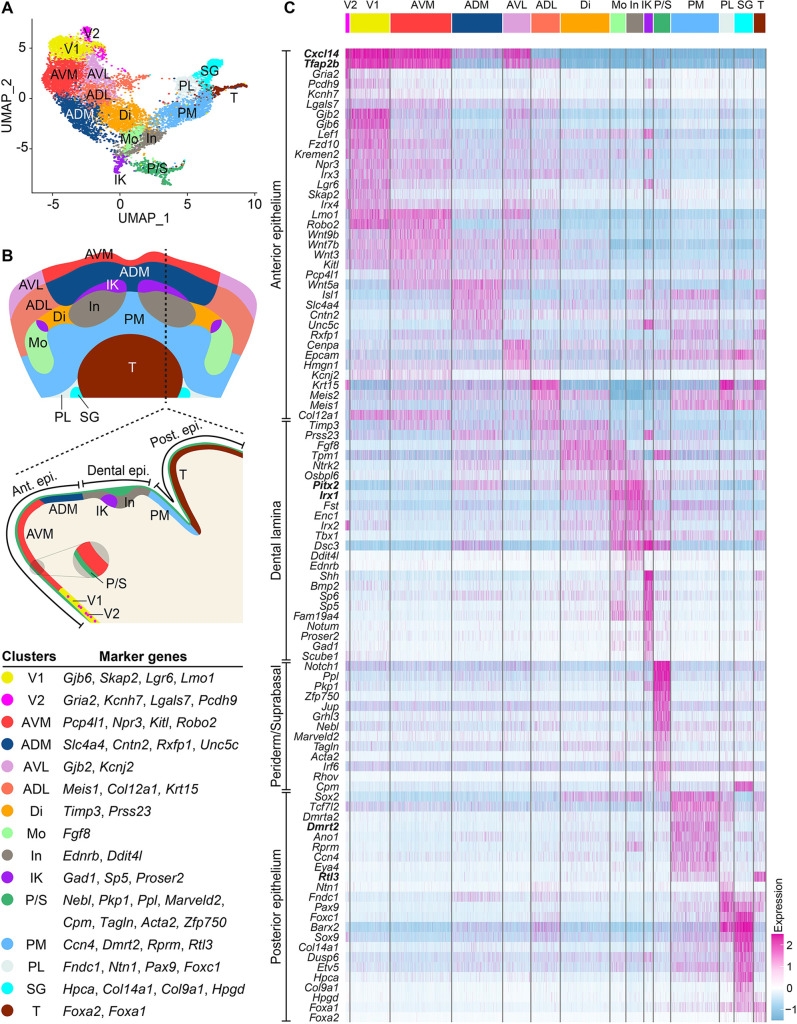
**Mandibular epithelial cells are clustered based on their anatomical positions.** (A) Second level UMAP clustering reveals 15 clusters. (B) Schematic drawings of the mandible in dorsal view and sagittal section showing the anatomical positions of the 15 clusters, which partition the anterior (ant.), dental and posterior (post.) epithelium (epi.) into subdomains. Representative markers used for *in situ* mapping are listed. (C) Heatmap of differentially expressed genes showing scaled expression level from low (blue) to high (pink). Genes in bold are markers for the broader epithelial regions indicated on the left. Clusters assigned: ADL, anterodorsal-lateral; ADM, anterodorsal-medial; AVL, anteroventral-lateral; AVM, anteroventral-medial; Di, diastema; IK, initiation knot; In, incisor; Mo, molar; PL, posterior-lateral; PM, posterior-medial; P/S, periderm and suprabasal cells; SG, salivary gland; T, tongue; V1, ventral 1; V2, ventral 2.

To obtain a more-detailed classification of the different subpopulations in the mandibular epithelium, we re-clustered the cells at a higher resolution. This yielded 15 clusters that are characterized by distinct gene expression signatures (Fig. 2). By identifying marker genes enriched in each cluster and mapping their spatial distribution within the epithelium, we were able to assign the identities of each cluster according to their anatomical positions: V1 (ventral 1), V2 (ventral 2), AVM (anteroventral-medial), AVL (anteroventral-lateral), ADM (anterodorsal-medial) and ADL (anterodorsal-lateral), which constitute cluster 1 described above; Di (diastema), In (incisor), Mo (molar) and IK (initiation knot), which subdivide cluster 2; PM (posterior-medial), PL (posterior-lateral), SG (salivary gland) and T (tongue) from cluster 3; and P/S (periderm and suprabasal cells), which makes up cluster 4. We further describe these clusters below.

### Anterior mandibular epithelium is patterned into subdomains and expresses regulators of WNT and BMP pathways

**Fig. 3.**
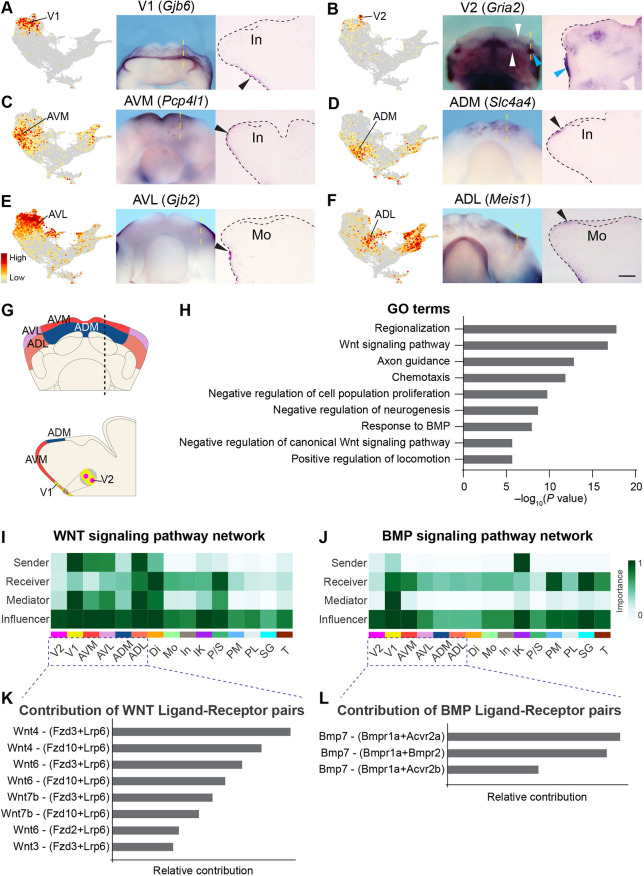
**Mapping clusters in the anterior mandibular epithelium.** (A-F) *Gjb6*, *Gria2*, *Pcp4l1*, *Slc4a4*, *Gjb2* and *Meis1* are markers for the indicated clusters. Their expression is shown in feature plots (left panels) and detected in E12.0 mandibles by whole-mount *in situ* hybridization (middle panels), viewed ventrally (A,B) or dorsally (C-F). Right panels are representative sagittal sections through the regions indicated by the yellow dashed lines; anterior is towards the left. Black arrowheads indicate epithelial expression on sections. *Gria2* is detected as puncta (white arrowheads) and in a lateral patch (cyan arrowheads). Black dashed lines outline the mandible and the dental epithelium. (G) Summary of cell groups in the anterior mandibular epithelium. (H) Bar graph showing enriched GO terms in the anterior clusters. (I,J) CellChat heatmaps showing the relative importance of each cluster in their WNT (I) or BMP (J) signaling roles. (K,L) Relative contribution of each ligand-receptor pair to the overall WNT or BMP signaling in the anterior mandibular epithelium. In, incisor; Mo, molar. Scale bar: 450 μm in whole-mount images in A,B; 280 μm in whole-mount images in C-F; 100 μm in cross-section images in A-F.

We first examined clusters corresponding to cluster 1 above, the epithelial region that extends from the ventral aboral mandible to the anterior of the dental lamina (Fig. 1A,E). Cluster V1 includes cells that occupy the ventral mandibular epithelium, based on the expression of its markers (Figs 2B and 3A, Fig. S3A-C). V2 shares a similar expression profile to V1 (Fig. 2C), but cells labeled by V2 markers are dispersed as puncta within the ventral epithelium, as well as in two lateral epithelial patches (Fig. 3B, Fig. S3D-F). We next studied cells from clusters AVM, ADM, AVL and ADL. Probing the expression of AVM and ADM markers (Fig. 2B) showed that these clusters contain cells in the medial region of the mandible that is anterior to the incisor (Fig. 3C,D, Fig. S3G-L). AVM cells are located in between ADM and V1 cells, consistent with their relative positions on the UMAP (Fig. 2A). Adjacent to the AVM and ADM clusters are AVL and ADL. Although the pairs of AVM/AVL and ADM/ADL share many transcriptional features (Fig. 2C), assessment of genes differentially expressed in AVL or ADL but reduced in AVM/ADM (Figs 2B and 3E,F, Fig. S3M-O) indicates that AVL and ADL cells are positioned in the lateral region of the anterior mandible (Fig. 3G). Therefore, although the *x*-axis of the UMAP corresponds to the oral-aboral axis of the mandible, the *y*-axis matches the medial-lateral axis. This is also consistent with the expression of known markers of the medial (e.g. *Bmp4*, *Isl1* and *Tlx1*) and lateral (*Fgf8*) regions of the mandible (Mitsiadis et al., 2003; Neubüser et al., 1997; Raju et al., 1993), labeling the bottom and the top halves of the UMAP, respectively (Figs S2E-H and S4J).

To probe for the functional importance of genes expressed in these clusters, we performed Metascape functional enrichment analysis. As the outputs from individual clusters were comparable, the anterior epithelial populations collectively perform similar functions, which are summarized in Fig. 3H. We found that there is an over-representation of genes related to WNT signaling, and the anterior epithelium is a major source of WNT ligands, expressing *Wnt3*, *Wnt4*, *Wnt5a*, *Wnt6*, *Wnt7a*, *Wnt7b*, *Wnt9b*, *Wnt10a* and *Wnt10b* (Tables S2 and S3). Several WNT inhibitors, *Axin2*, *Znrf3*, *Kremen2*, *Nkd1* and *Sostdc1*, are also upregulated, likely as a part of the negative feedback loop downstream of WNT signaling (Hao et al., 2012; Jho et al., 2002). In parallel, anterior epithelial cells are capable of mediating and modulating BMP signals, as they express *Msx1*, *Msx2*, *Nbl1* and *Htra1* (Chen et al., 1996; Hung et al., 2012; Launay et al., 2008). Finally, another functional category enriched in the anterior epithelium includes regulators of chemotaxis, such as *Ephb1*, *Ephb2*, *Robo2* and *Sema3a*, and they may contribute to axon guidance (Birgbauer et al., 2001; Evans et al., 2015; Nakamura et al., 2000) and/or the migration of other cell types in the developing mandible.

Analyzing cell-cell communications using CellChat (Jin et al., 2021) further highlighted the signaling functions of the anterior epithelium (Fig. S5), which can output WNT and influence BMP signals (Fig. 3I,J) to the underlying mesenchyme (Jin et al., 2011; Xu et al., 2019). Interestingly, CellChat identified many WNT and BMP ligand-receptor pairs within the anterior epithelium (Fig. 3K,L, Fig. S5E,F), suggesting that these signals could act locally to help specify and/or maintain the aboral epithelium.

### scRNA-seq identifies several novel markers for different tooth-related populations

**Fig. 4.**
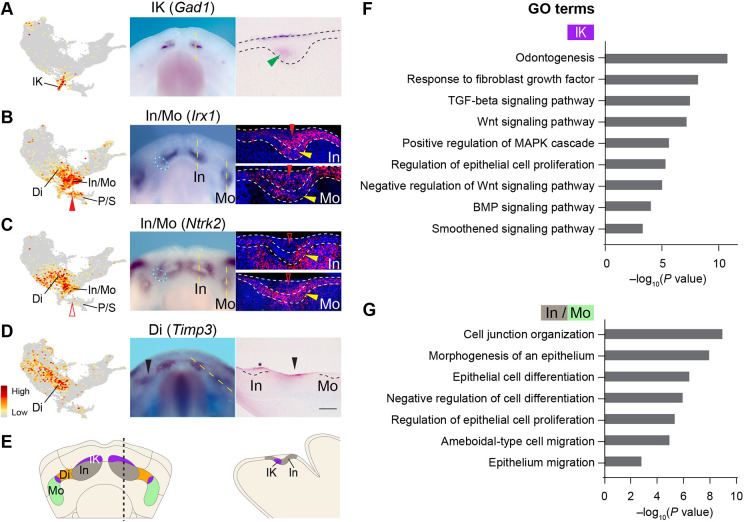
**Distinct cell populations in the dental lamina.** (A-D) Feature plots (left panels) and RNA *in situ* hybridization on E12.0 mandibles (middle panels, dorsal views) for markers enriched in dental-related clusters. Right panels show representative *in situ* (A,D) or RNAscope (B,C) staining on sections through the regions indicated by the yellow dashed lines; anterior is towards the left. Black and white dashed lines outline the dental epithelium. (A) *Gad1* labels the initiation knot (IK, green arrowhead). (B,C) *Irx1* and *Ntrk2* are expressed in the non-IK basal layer (yellow arrowheads) of the incisor (In) and the molar (Mo), and in parts of the diastema (Di, cyan dashed ovals). Solid and open red arrowheads indicate strong or weak expression, respectively, in the suprabasal cells, which are contained within the P/S (periderm/suprabasal) cluster. (D) *Timp3* labels the diastema (black arrowheads) and periderm cells over the incisor (asterisk). (E) Summary of dental-related clusters. (F,G) Bar graphs showing enriched GO terms in the IK (F) and In/Mo (G) clusters. Scale bar: 280 μm in whole-mount images in A-D; 50 μm in cross-sections in A-C; 100 μm in cross-section in D.

We next focused on clusters with dental signatures. Examining genes highly expressed in the IK cluster revealed known markers (*Shh*, *Dkk4* and *Fgf20*) of the tooth signaling center: the initiation knot (Fig. S4A) (Ahtiainen et al., 2016). We also validated several novel IK markers, including *Gad1*, *Sp5* and *Proser2* (Fig. 4A, Fig. S4B,C). As expected, the IK cluster is enriched with components of several signaling pathways that promote cell proliferation and tooth development (Fig. 4F, Fig. S5A-D).

Clusters In and Mo have similar transcriptional profiles (Fig. 2C) and express many dental markers, including *Pitx2, Irx1* and *Fst* (Mucchielli et al., 1997; Yu et al., 2017), as well as newly identified genes (e.g. *Ntrk2*, *Dsc3*, *Enc1* and *Osbpl6*), the expression of which we verified in the developing teeth (Fig. 4B,C, Fig. S4D-G). Further mapping using genes enriched in either cluster (Fig. 2B) assigned In and Mo to the incisor and the molar, respectively (Fig. S4H-J). Notably, many dental markers are expressed beyond the In and Mo clusters on the UMAP. For example, *Dsc3* and *Enc1* are additionally expressed in cluster IK, and their transcripts are present throughout the entire incisor bud at E12.0 (Fig. S4E,F). In contrast, *Irx1* and *Ntrk2* mRNAs are considerably lower in the IK cluster and cells expressing them occupy the non-IK region of the incisor bud (Fig. 4B,C). Many In and Mo (In/Mo) markers are also detected at high levels in cluster P/S, and this corresponds to their expression in both the basal and suprabasal layers of the tooth bud (e.g. *Irx1* and *Dsc3* in Fig. 4B and Fig. S4E). In contrast, In/Mo markers with minimal presence in cluster P/S are localized primarily to only the basal layer (e.g. *Ntrk2* in Fig. 4C). Therefore, cells in clusters In/Mo most likely represent the dental basal cells, while suprabasal cells are transcriptionally closer to the peridermal cells in cluster P/S, which we describe later. At the functional level, many of the In/Mo genes are involved in regulating proliferation and differentiation (Fig. 4G), thus consistent with the general role of an epithelial basal layer, where progenitor cells divide and give rise to more differentiated suprabasal cells (Lechler and Fuchs, 2005). Interestingly, regulators of cell protrusions and adhesions, such as *Slitrk6*, *Wasf1*, *Dock5*, *Flrt3* and *Ednrb* are specifically upregulated in In/Mo cells (Tables S2 and S3) and they may control the basal-to-suprabasal delamination process (Frank et al., 2017; Kasioulis et al., 2022; Katayama et al., 2009; Pla and Larue, 2003; Seiradake et al., 2014).

Last, we examined the expression of Di markers *Timp3* and *Prss23*, which maps Di to the diastema, a space between the incisor and molar buds (Fig. 4D and Fig. S4K). *Irx1* and *Ntrk2* are also expressed in parts of Di on the UMAP, and their mRNA expression correspondingly extends into the diastema (cyan ovals in Fig. 4B,C). The assignment of tooth-related clusters is summarized in Fig. 4E.

### Suprabasal populations are diverse and exhibit transcriptional features for strong cell-cell adhesion and cell movement

**Fig. 5.**
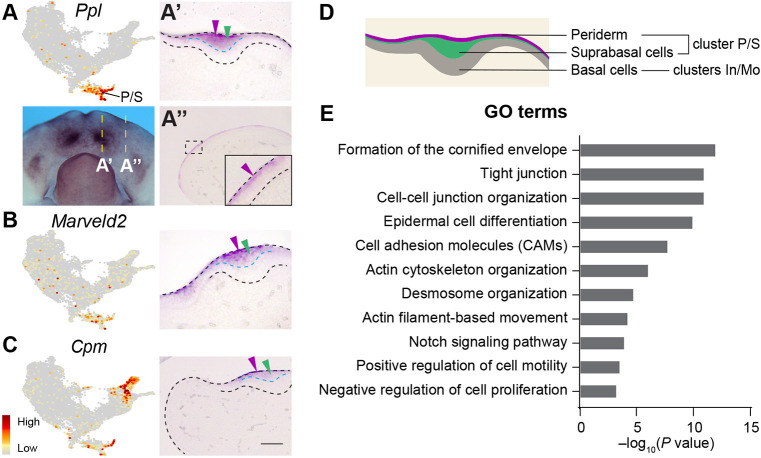
**Characterization and functional enrichment of peridermal and suprabasal (P/S) genes.** (A-C) Feature plots show P/S markers. *In situ* hybridization on sagittal sections (anterior towards the left) show their expression in the periderm (purple arrowheads) and the suprabasal cells (green arrowheads) of the incisor bud, but not in the basal layer (below the cyan dashed lines). (A′,A″) Sections taken at the regions indicated by the yellow dashed lines. Black dashed lines outline the mandible and the incisor. (D) Schematic of a dental placode showing different populations and their clusters. (E) Bar graph showing enriched GO terms in cluster P/S. Scale bar: 280 μm in A; 50 μm in A′,B,C; 100 μm in A″; 25 μm A″ (inset).

Our analysis so far indicates that cluster P/S contains peridermal and suprabasal cells. To verify this, we examined the expression of P/S-specific markers (Fig. 2B) and found that they indeed label both cell types, but are excluded from the basal layer (Fig. 5A-D). Functional enrichment analysis revealed that P/S cells are characterized by tight junction and desmosome genes (e.g. *Cldn4*, *Jup* and *Ppl*) (Fig. 5E, Table S3), which are often associated with differentiating cells in stratified epithelia (Koster and Roop, 2007). At the signaling level, Notch signals are prevalent in the P/S (Figs S5A-D and S6A), supporting the idea that Notch signaling controls periderm and suprabasal cell generation (Blanpain et al., 2006; Casey et al., 2006). Finally, P/S cells are enriched with factors that organize actin cytoskeletons and promote cell motility (e.g. *Limk2*, *Pdlim5*, *Csrp1*, *Myl9* and *Rhov*) (Fig. 5E). This would enable cell movement and produce forces needed for tooth invagination as previously discovered (Panousopoulou and Green, 2016).

To dissect the heterogeneity among P/S cells, we next performed sub-clustering and identified four sub-populations (P/S1-4) (Fig. S6B-K, Table S4). Intriguingly, P/S1-3 match the oral-aboral patterning we have observed in the mandible, expressing the same positional markers that respectively label the aboral (P/S1, Fig. S6D,E), dental (P/S2, Figs S4E,K and S6F) and posterior oral (P/S3, Fig. S6G-I) epithelium. P/S1-3 therefore comprise peridermal and suprabasal cells that retain positional identities. P/S4 contains a peridermal subtype population that disperses over the epithelial surface and expresses the recently identified markers *Tagln* and *Acta2* (Fig. S6J,K). The transcriptional heterogeneity among P/S cells thus reflect differences in their localizations and subtypes.

### E12.0 tongue epithelium is a transcriptionally distinct population that includes precursor cells of taste bud primordia

**Fig. 6.**
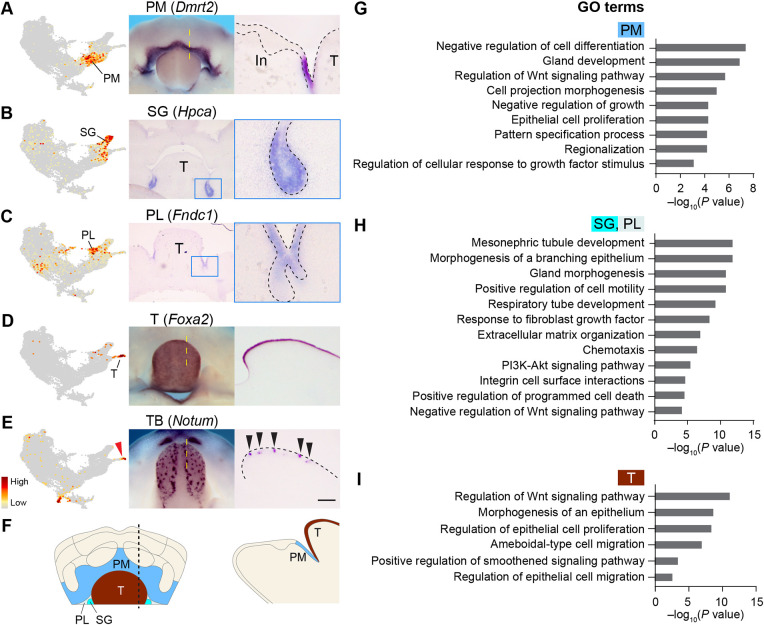
**Mapping the posterior mandibular clusters.** (A-D) Left panels show feature plots of indicated markers for posterior clusters. Middle panels show *in situ* hybridization on E12.0 whole mandibles (A,D, dorsal views) or frontal sections (B,C). Right panels are sagittal sections (anterior towards the left) taken at the regions indicated by the yellow dashed lines in A,D or by the rectangles in B,C. (E) The tongue cluster contains taste bud (TB) precursor cells (red arrowhead), here detected using *Notum* in E12.5 taste bud placodes (black arrowheads). (F) Summary of the posterior mandibular epithelial populations. (G-I) Graphs showing enriched GO terms in the posterior clusters. In, incisor; PL, posterior-lateral; PM, posterior-medial; SG, salivary gland; T, tongue. Scale bar: 280 μm in middle panels of A,D,E; 200 μm in middle panels of B,C; 50 μm in right panels of A-C; 100 μm in right panels of D,E.

The rest of the UMAP contains epithelial cells posterior to the dental tissues, encompassing clusters PM, PL, SG and T. Cluster PM expresses *Dmrt2*, *Ccn4* and *Rprm*, which are mapped to the space between the tooth and the tongue (Fig. 6A,F, Fig. S7A,B). PM cells also express genes known to maintain the progenitor state (e.g. *Meis1*, *Sox2* and *Smarca2*) (Bhattacharya et al., 2019; Centore et al., 2020; Okumura et al., 2014) (Fig. 6G, Table S3), which could function here to limit epithelial differentiation and stratification. Cluster SG represents the salivary gland bud (Fig. 6B, Fig. S7D-F), while cluster PL includes junctional cells lateral to the tongue that connect the salivary gland to the mandibular and tongue epithelium (Fig. 6C, Fig. S7G-I). Functional enrichment analysis showed that SG and PL are enriched with regulatory genes important for branching morphogenesis in ductal organs (e.g. *Six1*, *Six2* and *Foxc1*) (Fig. 6H, Table S3) (Laclef et al., 2003; Mattiske et al., 2006). In parallel, the upregulation of genes associated with FGF signaling and apoptosis is concordant with the developmental process of salivary gland lumen formation (Patel et al., 2011).

Cluster T contains tongue epithelial cells, marked by *Foxa1* and *Foxa2* (Fig. 6D, Fig. S7C). Curiously, *Shh*, a known marker of the forming taste buds (Thirumangalathu et al., 2009), labels the tip of cluster T on the UMAP (Fig. S8B). We therefore performed sub-clustering and identified additional markers for this subpopulation (Table S5), which are expressed in the taste bud primordia (Fig. 6E, Fig. S8A-D). Interestingly, taste bud and dental placodes co-express several markers (Fig. S8E), indicative of similar developmental processes and signaling regulations (Figs 4F,G and 6I).

### Mandibular epithelium undergoes progressive regionalization along the oral-aboral axis

**Fig. 7.**
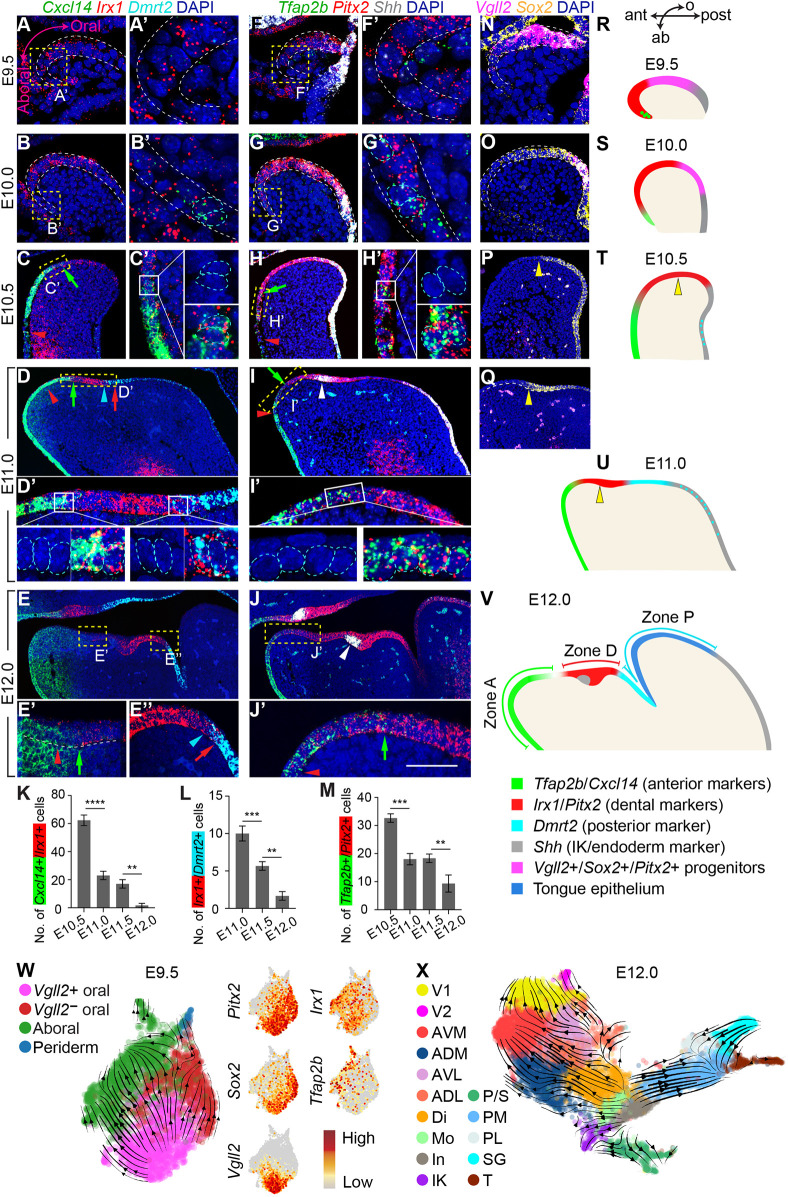
**Transcriptional regionalization of the mandibular epithelium along the oral-aboral axis.** (A-J′) RNAscope analysis showing spatiotemporal expression changes of markers for the anterior mandibular epithelium (*Cxcl14* and *Tfap2b*, green), the dental epithelium (*Irx1* and *Pitx2*, red), the posterior epithelium (*Dmrt2*, cyan) and the oropharyngeal endoderm (*Shh*, white) from E9.5 to E12.0. *Shh* also labels the initiation knot (white arrowheads in I,J). Representative sagittal sections through the presumptive incisor region (A-D′,F-I′) or the incisor (E-E″,J,J′) are shown; anterior towards the left. White dashed lines outline the epithelium. (A′-J′) Enlargements of the areas outlined in yellow in A-J; white boxes in C′,D′,H′,I′ show further close-ups. Cyan dashed lines outline cells co-expressing different zonal markers*.* Colored (red, green and cyan) arrowheads and arrows, respectively, mark the anterior and posterior limit of cells labeled by the same color, denoting the spread of boundary cells with overlapping marker expression. (K-M) Quantification of boundary cell numbers (*n*=3 embryos). (N-Q) *Vgll2* and *Sox2* expression from E9.5 to E11.0. Yellow arrowheads indicate the anterior *Sox2* expression border. (R-V) Schematics summarizing the expression pattern of regional markers and the establishment of different epithelial zones along the oral(o)-aboral(ab) and anterior(ant)-posterior(post) axes. (W,X) RNA velocity showing predicted epithelial lineages in E9.5 and E12.0 mandibles. IK, initiation knot; zone A, anterior zone; zone D, dental zone; zone P, posterior zone. Scale bar: 60 μm in A,C′,D′,F,H′,I′,N; 100 μm in B,E′,E″,G,J′,O; 200 μm in C-E,H-J,P,Q; 20 μm in A′,B′,F′,G′ and white boxes in C′,D′,H′,I′. Data are presented as mean±s.d. *P*-values were determined using one-way ANOVA and Tukey's HSD test (**P<0.01; ***P<0.001; ****P<0.0001).

Our analyses so far have established the spatial pattern of different mandibular epithelial populations at E12.0. The identification of several region-specific markers provided an opportunity to study how the tooth and its neighboring epithelium are patterned along the oral-aboral axis over time, which remains not well understood. At E12.0, *Irx1* and *Pitx2* are robust markers of the developing teeth (Figs 1F and 4B). Based on the UMAP, the *Irx1*^+^ and *Pitx2*^+^ dental domain should in theory be bounded anteriorly by cells in the ADM, AVM, ADL and AVL clusters that collectively express *Cxcl14* and *Tfap2b* and be bounded posteriorly by *Dmrt2*-expressing PM cells (Fig. 2). To simultaneously visualize these markers and compartments, we performed RNAscope *in situ* hybridization on sagittal sections at the level of the incisor bud. Using the combination of *Cxcl14*, *Irx1* and *Dmrt2*, and *Tfap2b*, *Pitx2* and *Shh*, where *Shh* is a marker for the initiation knot, we show that at E12.0 the mandibular epithelium is divided into three main zones: the zone anterior to the dental epithelium (zone A); the dental zone (zone D); and the zone posterior to the tooth (zone P) (Fig. 7E,J,V). At the inter-zone junctions, cells co-express markers from the neighboring regions. For example, *Cxcl14^+^* and *Irx1^+^*, and *Irx1^+^* and *Dmrt2^+^* cells, respectively, span the A/D and D/P boundaries, which at this stage consist of two to four cells (Fig. 7E′,E″,K,L). The *Tfap2b^+^* and *Pitx2^+^* A/D boundary is comparably wider (Fig. 7J′,M).

To examine how the spatial pattern of these markers change during mandibular development, we analyzed the expression of the same set of genes in younger embryos between E9.5 and E11.5. At these stages, *Shh* is a marker for the oropharyngeal endoderm (Haworth et al., 2007) and helps to delineate the expression of other markers in the ectoderm. Lineage tracing with *Shh^CreER^*;*R26^mT/mG^* showed that *Shh^+^* endodermal descendants are mostly restricted to the posterior tongue (Fig. S9A), supporting results from *Sox17-2A-iCre* mice (Rothova et al., 2012). At E9.5, when the mandibular epithelium is only a monolayer, zone D markers *Irx1* and *Pitx2* are present in the majority of ectodermal cells and border the *Shh*+ endoderm (Fig. 7A,A′,F,F′,R, Fig. S10A-C). In comparison, whereas the zone A marker *Tfap2b* is expressed in the ventral-lateral arch (Fig. S10C), *Cxcl14* and *Dmrt2* are barely detectable at this stage. At E10.0, zone A formation proceeds in the ventral-medial arch, as *Irx1*^+^ and *Pitx2*^+^ cells next to the developing heart now also begin to express both *Cxcl14* and *Tfap2b* (Fig. 7B,B′,G,G′,S). Their expression then becomes noticeably expanded between E10.0 and E10.5 (Fig. 7C,H,T). *Irx1* and *Pitx2* expression is now located at the oral surface (Fig. 7C,H, Fig. S10D), but boundary cells co-expressing *Cxcl14* and *Irx1* or *Tfap2b* and *Pitx2* remain broadly distributed (Fig. 7C′,H′,K,M). Posteriorly, *Irx1* and *Pitx2* continue to adjoin the *Shh*^+^ endoderm (Fig. 7C,H,T). By E11.0 *Dmrt2*^+^ cells finally emerge in the ectoderm between the dental lamina and the endoderm, encompassing the region that would form zone P and the anterior part of the tongue (Fig. 7D,I,U, Fig. S9B,C). We made a similar observation using another posterior marker: *Rtl3* (Fig. S9D,E). The P/D boundary is thus established at E11.0 and delineates *Irx1* in the dental lamina posteriorly. Both A/D and P/D boundaries continue to narrow thereafter, such that from E10.5 to E12.0 these boundaries are progressively sharpened (Fig. 7C′-E″,H′-J′,K-M), just as the three zones become increasingly defined (Fig. 7T-V).

Oral-aboral patterning therefore begins as early as E9.5, when *Tfap2b*-labeled aboral cells can be discerned from *Pitx2*-labeled oral cells in the lateral arch (Fig. S10C). To explore how these cells first arise, we conducted RNA velocity (Bergen et al., 2020) following scRNA-seq of E9.5 mandibles. Four epithelial clusters are distinguishable at this stage: *Pitx2^+^* and *Sox2*^+^ oral populations that differ in *Vgll2* expression; *Tfap2b*^+^ aboral cells; and *Acta2^+^* and *Tagln*^+^ peridermal cells (Fig. 7W, Fig. S10G). The velocity field projected onto the UMAP begins from a source of *Vgll2*^+^, *Pitx2*^+^ and *Sox2^+^* cells. It then flows through *Pitx2*^+^ and *Irx1*^+^ populations across clusters and ends at *Tfap2b*-expressing aboral cells, which still express *Irx1* at this stage (Fig. 7W). Zone A founder cells thus likely form from zone D precursors that originate from the *Vgll2*^+^, *Pitx2*^+^ and *Sox2^+^* progenitor cells. These progenitors would also produce *Vgll2*-negative oral cells and the periderm, coinciding with stratification initiation.

Consistent with the RNA velocity analysis, at E8.5 *Vgll2*, *Pitx2* and *Sox2* are co-expressed by cells in the dorsal two-thirds of the nascent mandibular arch epithelium, while *Irx1* has a ventrally biased expression and *Tfap2b* is undetectable (Fig. S10H). Patterning thus has begun in a few ventral cells at E8.5. As epithelial zones expressing dental *Pitx2* and *Irx1* and aboral *Tfap2b* and *Cxcl14* markers sequentially arise and expand between E8.5-E10.5, *Vgll2*, *Pitx2* and *Sox2* co-expression is momentarily maintained in the posterior epithelium (Fig. 7A-B′,F-G′,N,O). *Vgll2* eventually disappears around E10.5 (Fig. 7P), preceding zone P appearance and zone D confinement to the anterior *Sox2* border (Fig. 7D,I,Q,U). Narrowing of zone D is unlikely due to differential expansion of neighboring regions, as oral epithelial proliferation appears uniform, while aboral cells in effect have reduced proliferation (Fig. S10E,F).

Once zones A and D are fully established at E12.0, we noticed that their marker expression at the A/D boundary becomes considerably reduced. Because this is where ADM cells are located, we reasoned that the ADM fate is newly specified around E12.0. To test this, we compared our E12.0 scRNA-seq data with a published E10.5 dataset (Xu et al., 2019). This revealed that most zone A cells at E10.5 are clustered within V1, AVM and AVL (Fig. S9F,G) and that ADM cells would form at a later stage. Concordantly, the ADM marker *Rxfp1* is not expressed in the anterior-dorsal region until E12.0 (Fig. S9C,E). Cluster PM cells also form in such a sequential manner, emerging after the formation of zones A and D (Fig. 7C,D, Fig. S9F,G). Notably, RNA velocity showed cell flows from zone D towards ADM and PM, suggesting that, like E9.5 ectoderm, zone D begets neighboring regions (Fig. 7X). Together, these results demonstrated that mandibular oral-aboral patterning is a dynamic regionalization process that accompanies its growth. As the epithelium expands from the original *Vgll2*^+^, *Pitx2*^+^ and *Sox2^+^* populations, their descendants in the anterior and posterior mandible progressively adopt regional identities between E9.5 and E12.0, such that the initial broad expression of *Irx1* and *Pitx2* is eventually delimited to the developing tooth.

### Region-specific transcription factors underlie the transcriptional differences between epithelial populations

**Fig. 8.**
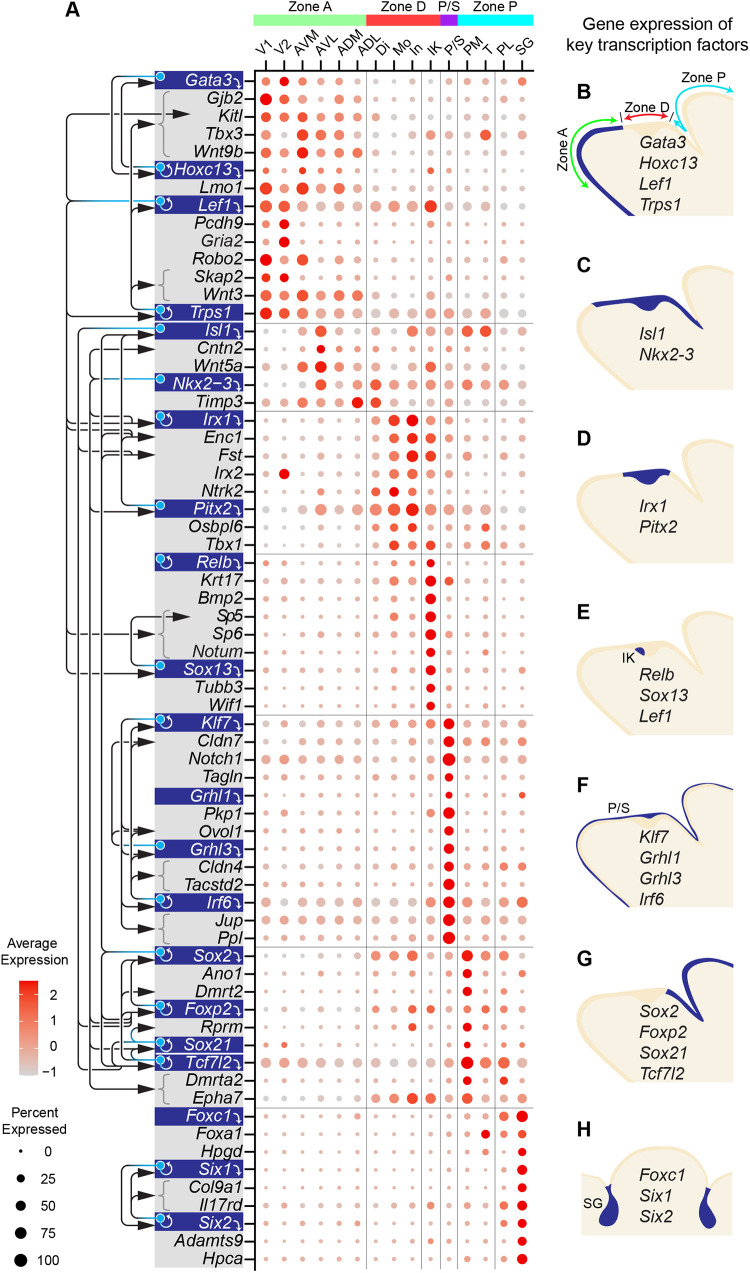
**SCENIC reveals population-specific transcription factors and downstream targets.** (A) Dot plot showing the expression of genes encoding key transcription factors (blue background) and examples of their putative targets (gray background below downward white arrows, with additional links indicated by black arrows). (B-H) Schematics of sagittal sections through the incisor (B-G) or a frontal section through the submandibular glands (H) depicting the RNA expression of key transcription factors. ADL, anterodorsal-lateral; ADM, anterodorsal-medial; AVL, anteroventral-lateral; AVM, anteroventral-medial; Di, diastema; IK, initiation knot; In, incisor; Mo, molar; PL, posterior-lateral; PM, posterior-medial; P/S, periderm and suprabasal cells; SG, salivary gland; T, tongue; V1, ventral 1; V2, ventral 2; zone A, anterior zone; zone D, dental zone; zone P, posterior zone.

To gain insight into the gene regulatory network (regulon) that defines mandibular epithelial populations, we applied the SCENIC pipeline (Aibar et al., 2017) on our E12.0 dataset to computationally infer key transcription factors and their target genes based on co-expression and enrichment of *cis*-regulatory motifs. This yielded region- and cluster-specific regulons (Fig. 8 shows curated interactions; full lists in Table S6), which include many of the cluster-defining markers (Fig. S11). For example, *Gata3*, *Hoxc13*, *Lef1* and *Trps1* encode transcription factors differentially expressed in the anterior/aboral epithelium, and many anterior markers contain their putative binding sites (Fig. 8A,B). The detection of a LEF1 regulon echoes our findings that active WNT signaling is a key feature of the anterior/aboral epithelium (Fig. 3H,I). Also notably, TRPS1 is a context-dependent transcriptional repressor/activator that recognizes the GATA-binding sequence (Wang et al., 2018; Fantauzzo and Christiano, 2012). As TRPS1 and GATA3 regulons share several targets (Fig. 8A) and their mutations affect mandibular and tooth development (Abe et al., 2021; Malik et al., 2002), they may modulate the expression of a gene set to help specify the aboral mandible. In contrast, *Nkx2-3* is expressed in the oral epithelium (Fig. 8A,C). It targets *Irx1*, *Pitx2*, *Foxp2* and *Sox21*, all of which are transcription factors with their own regulons (Fig. 8A). IRX1 and PITX2 are the predicted main transcription factors in the dental epithelium (Fig. 8A,D, Fig. S12A), while FOXP2 and SOX21, together with SOX2 and TCF7L2, target genes in the posterior oral epithelium (Fig. 8A,G, Fig. S12D).

Our SCENIC analysis also uncovered regulons by RELB, LEF1 and SOX13 in the initiation knot (Fig. 8A,E and Fig. S12B). RELB is a transcription factor within the NF-κB pathway and could function downstream of the Eda and Edar signaling to control the initiation knot size (Ahtiainen et al., 2016). Concurrently, LEF1 would support the proposed WNT signaling function in promoting initiation knot formation (Mogollón et al., 2021). In the P/S, we identified regulons associated with GRHL1, GRHL3 and IRF6 (Fig. 8A,F), which are crucial for periderm differentiation (de la Garza et al., 2013; Miles et al., 2017; Peyrard-Janvid et al., 2014). They target several tight junction and desmosome components (Fig. 8A and Fig. S12C), many of which also contain motifs for KLF7, a Krüppel-like transcription factor. Last, FOXC1, SIX1 and SIX2 are key transcription factors for the salivary gland (Fig. 8A,H, Fig. S12E). As these factors are required for the development of different glandular organs (Laclef et al., 2003; Mattiske et al., 2006), they may initiate a common regulatory program to direct gland morphogenesis. Together, our analysis revealed multiple gene regulatory networks that define distinct mandibular epithelial populations.

### NTRK2 promotes epithelial invagination during early tooth development

As our E12.0 scRNA-seq data successfully revealed genes enriched in different oral epithelial appendages, it provides a useful platform to uncover novel regulators of epithelial morphogenesis. As a proof of principle, we focused on the developing tooth and its newly characterized marker *Ntrk2*, which encodes the neurotrophic receptor tyrosine kinase 2. NTRK2 is a receptor for brain-derived neurotrophic factor (BDNF) and neurotrophin 5 (NTF5), and its signaling activation regulates proliferation and differentiation in other contexts (Bartkowska et al., 2007; Li et al., 2008). Because cell proliferation is important for early tooth morphogenesis (Li et al., 2016a), we tested whether signaling via NTRK2 promotes tooth growth.

We began our analysis by first examining the spatiotemporal expression of *Ntrk2* in more detail using RNAscope. Whereas few *Ntrk2* transcripts were observed in the dental epithelium before E11.5, robust expression was detected in the non-IK basal layer and the adjacent suprabasal and mesenchymal cells at E12.0 (Fig. 4C, Fig. S13). *Bdnf* is also expressed in the dental epithelium at E12.0 (Fig. S13B), indicating that BDNF could locally signal to NTRK2 at this stage.

**Fig. 9.**
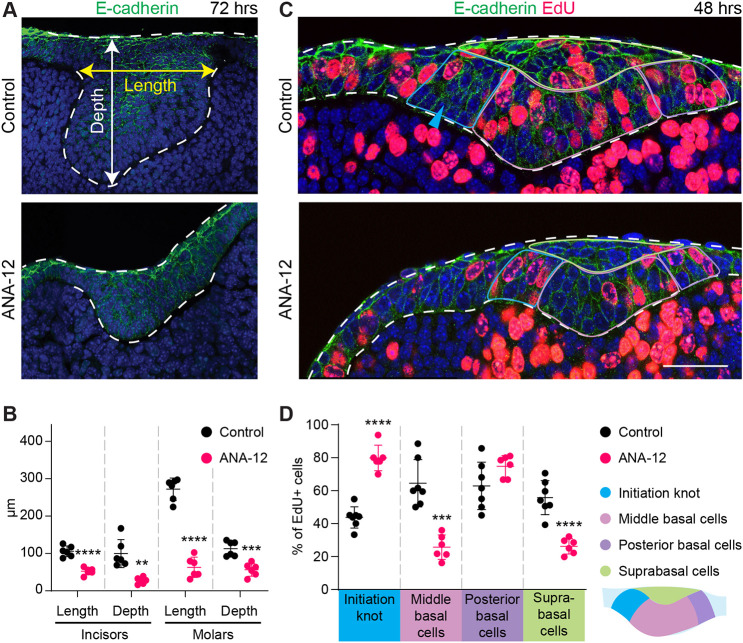
**NTRK2 signaling promotes dental epithelial growth and invagination.** (A) Sagittal sections through the incisor epithelium of E11.5 mandible explants cultured for 72 h in DMSO (control) or with the NTRK2 inhibitor ANA-12; anterior towards the left. (B) Quantification of the length and depth (as shown in A) of control or ANA-12-treated tooth germs (*n*=6). (C) EdU staining on sagittal sections of E11.5 incisors treated with DMSO or ANA-12 for 48 h; anterior is towards the left. The four sub-regions used for quantifying EdU^+^ cells are indicated. Cyan arrowhead marks the initiation knot. (D) Quantification of EdU^+^ cells in the control (*n*=7 embryos) or ANA-12-treated (*n*=6 embryos) incisor epithelium. Dashed lines outline the epithelium, labeled using E-cadherin antibody. Scale bar: 80 μm in upper panel in A; 50 μm in lower panel in A; 30 μm in C. Quantitative data are presented as mean±s.d. *P*-values were determined using an unpaired Student's *t*-test (**P<0.01; ***P<0.001; ****P<0.0001).

To understand NTRK2 function during tooth development, we cultured E11.5 mandible explants in the presence or absence of ANA-12, a selective NTRK2 antagonist (Cazorla et al., 2011). This would block NTRK2 signaling at the onset of its expression and before the rapid growth of a dental placode. After 3 days of culture, ANA-12-treated samples had significantly smaller tooth buds than controls (Fig. 9A,B). EdU-labeling of cycling cells showed that there were significantly fewer proliferating cells in the suprabasal layer and in the middle region of the basal layer in ANA-12-treated incisor buds after 2 days of culture (Fig. 9C,D). NTRK2 inhibition also affected initiation knot maturation, as ANA-12-treated incisors failed to upregulate the cell cycle inhibitor p21 in the initiation knot, preventing the cell cycle arrest typically observed there (Fig. 9C,D, Fig. S14) (Du et al., 2017). Proliferation remained largely unchanged in the mesenchyme and in molar germs, indicating that NTRK2 function is tooth type specific, and it may control molar morphogenesis through mechanisms independent of proliferation. Together, these results revealed a novel function for NTRK2 in promoting cell proliferation and tissue growth in the incisor.

## DISCUSSION

Our study unveiled the transcriptional profiles that define each of the populations that make up the oral epithelium at E12.0. The resulting atlas not only complements existing knowledge of genes expressed in specific oral structures but also extends previous efforts to profile cells in the developing mandible at other developmental stages (Landin et al., 2012; Musselmann et al., 2011; Hauser et al., 2020; Yuan et al., 2020; Xu et al., 2019). Our data highlight the spatial and temporal gene expression changes that reflect the oral-aboral patterning of the mandibular epithelium. Furthermore, we have identified the gene regulatory networks in different populations that may play key roles in regulating cell fates and functions. Together, this study provides a catalog of epithelial cell types in the developing mandible and offers a resource for further investigation into the function of specific genes or pathways during epithelial morphogenesis.

### Regulation of the mandibular epithelium by WNT

Signaling interactions between the oral epithelium and the underlying mesenchyme, as well as within the epithelium itself, are crucial for the development of oral ectodermal structures (Balic and Thesleff, 2015). Although our results emphasize many of these signaling processes, the anterior aboral epithelium is an underappreciated signaling region identified here as a major source of WNT ligands. As WNT9b from the aboral epithelium has been shown to promote mesenchymal cell proliferation and survival at E10.5 (Jin et al., 2020), anterior WNTs at E12.0 may function similarly to enable further mandibular outgrowth. Within the oral epithelium, WNT/β-catenin signaling is a key regulator of tooth formation, as its inactivation abrogated tooth development (Liu et al., 2008; Sasaki et al., 2005) and hyperactivation led to supernumerary teeth (Järvinen et al., 2006; Wang et al., 2009). However, WNT responsiveness is not restricted to the dental epithelium, as the expression of the WNT target gene *Axin2* and WNT activity reporters both indicate active WNT signaling in the aboral epithelium (Lohi et al., 2010; Van Otterloo et al., 2022). This is consistent with the identification of WNT ligand-receptor pairs and the LEF1 regulon in these cells. Yet teeth are normally not formed at the ventral mandible. Therefore, an important question to consider here is how do mandibular epithelial cells interpret WNT signals to become different cell types? Paradoxically, although ectodermal deletion of *Tfap2a* and *Tfap2b* downregulated WNTs, it also induced the formation of ectopic incisors at the ventral surface of mutant mandibles (Woodruff et al., 2021; Van Otterloo et al., 2022). The ability to form teeth may thus be modulated by a balance between WNT activity and a yet to be identified inhibitory mechanism downstream of *Tfap2a* and *Tfap2b*. We also noticed that several WNT inhibitors are more highly expressed in the aboral epithelium than in the dental epithelium and could further modulate WNT activities in some regions.

Another possible mechanism for diversifying WNT responses is by employing different transcription factors. We found that *Tcf7l2* is differentially expressed in the posterior oral epithelium and targets many PM markers. Although LEF1 functions as a transcription activator under high WNT activity, TCF7L2 binds to the same target sites under low WNT signal and can act as an activator or repressor in a context-dependent manner (Korinek et al., 1997; Guo et al., 2021). As the posterior mandible is further away from the anterior WNT source, a different WNT-induced expression profile via TCF7L2 can be generated.

### Patterning of the mandibular epithelium along the oral-aboral axis

In order to understand how different oral structures form in the right place and at the right time, we must first characterize how the developing mandible is patterned. By mapping the expression of region-specific markers identified from our scRNA-seq analysis at different developmental timepoints, we demonstrated that the mandibular epithelium is progressively subdivided into distinct zones along the oral-aboral axis between E8.5 and E12.0. During early mandibular development, *Vgll2*^+^, *Pitx2*^+^ and *Sox2^+^* ectodermal cells first give rise to a group of broadly distributed cells expressing *Pitx2* and *Irx1*, which are dental markers at E12.0 and are predicted to be the main transcription factors driving the expression of other tooth-specific genes. Notably, markers related to the further maturation and growth of the tooth germ, such as *Ntrk2* and *Dsc3*, are not expressed until after E10.5 (Figs. S13 and S15). This suggests that the *Pitx2*^+^ and *Irx1*^+^ epithelium at E9.5 and E10.0 is transcriptionally competent for forming dental cells but has yet to initiate the full dental program. *Pitx2* and *Irx1* expression later becomes progressively confined first to the oral surface and then to the forming dental lamina, as ventral and anterior *Pitx2*^+^ and *Irx1*^+^ cells are patterned into *Tfap2b*^+^ and *Cxcl14*^+^ aboral populations, and the posterior epithelium adopts the *Dmrt2*^+^ and *Rtl3*^+^ identity. We propose that the mandibular ectoderm initially develops along a trajectory towards the dental fate, but subsequent specification of anterior and posterior cells with respective regional identities gradually delineates the boundary of the maturing tooth field and confines the dental lamina to its position along the oral-aboral axis. In the absence of correct patterning, as in the *Tfap2a* and *Tfap2b* mutants mentioned above, the aboral epithelium retains its tooth-forming capability and develops ectopic teeth (Woodruff et al., 2021). Our model can also be reconciled with current ideas of tooth evolution, where its origin – regardless of an ectodermal or endodermal root – is thought to arise by first adopting a genetic program that is competent to form placodes (Fraser et al., 2010), perhaps similar to the observed state at E9.5. The position and the final specification of the dental fate depend on how the neighboring epithelium is patterned thereafter, shifting the location of teeth or tooth-like structures along the oropharyngeal-aboral axis during evolution (Chen et al., 2020).

The process of epithelial regionalization we have observed in mouse mandibles is accompanied by the establishment and gradual sharpening of expression boundaries between markers labeling adjacent zones. How these boundaries are formed and regulated in the mandibular epithelium is not understood. The juxtaposition of *Wnt7b* in the anterior epithelium and *Shh* in the dental epithelium has been proposed to determine the boundary position (Sarkar et al., 2000). In other developing tissues with gene expression boundaries, such as the vertebrate hindbrain and the *Drosophila* wing, the boundary sharpness is enhanced through mutual repression of transcription factors downstream of morphogen-directed tissue patterning (Krumlauf and Wilkinson, 2021; Sokolowski et al., 2012; Yu and Small, 2008). Among the cluster-specific transcription factors we identified from the regulon analysis, several of them, including *Trps1*, *Irx1*, *Tcf7l2* and *Six1*, have context-dependent repressor functions (Wang et al., 2018; Cavodeassi et al., 2001; Brugmann et al., 2004; Korinek et al., 1997). It will be informative in the future to examine their reciprocal regulation.

### NTRK2 as a regulator of early tooth morphogenesis

Our dataset serves as a useful resource to study novel regulators of epithelial morphogenesis. Using the developing tooth as a model, we were interested in understanding the function of *Ntrk2* because of its unique expression pattern. Although previous studies have implied a role in tooth innervation, based on low-resolution expression analysis of *Ntrk2* and *Bdnf* in rats (Iwamoto et al., 2011; Luukko et al., 1996, 1997), whether NTRK2 signaling can regulate tooth morphogenesis was never addressed. Using the selective NTRK2 antagonist ANA-12, we were able to show for the first time that signaling through NTRK2 promotes tooth invagination. In the incisor, this is in part through the ability of NTRK2 to promote epithelial cell proliferation, which is crucial for generating suprabasal cells and thickening the placode (Li et al., 2016a). Although we could not rule out indirect regulation through mesenchymal NTRK2, expression of both *Bdnf* and *Ntrk2* in the dental epithelium suggests that direct BDNF and NTRK2 signaling can take place there. Whether NTRK2 is essential for the eventual tooth formation requires further investigation, especially because odontogenesis is a robust process and other mutants with reduced epithelial proliferation can still form teeth (Liu et al., 2015, 2019). Although tooth phenotypes have not been reported in *Ntrk2* null mice, most of which die shortly after birth (Klein et al., 1993; Rohrer et al., 1999), analyzing their tooth size and shape will be an important next step. Finally, NTRK2 may modulate tooth morphogenesis via other mechanisms. For example, TrkB-T1, the truncated form of NTRK2 lacking the kinase domain, can signal through small Rho GTPases to regulate cell shape and migration (Li et al., 2009; Ohira et al., 2005). Given the presence of TrkB-T1 in the dental epithelium (Iwamoto et al., 2011), it is conceivable that NTRK2/TrkB-T1 additionally promotes cell movement to propel the invagination process.

Taken together, our results have unveiled all the epithelial cell types in the developing mandible and describe the spatiotemporal distribution of key markers. This work provides a valuable resource for investigating mandibular patterning and morphogenesis, and offers a transcriptional roadmap to help future derivation of different oral epithelial progenitors for tissue bioengineering and regenerative medicine.

## MATERIALS AND METHODS

### Mouse lines, colony maintenance and procedures

*K14^Cre^* (Dassule et al., 2000), *R26^mT/mG^* (Muzumdar et al., 2007), *Shh^CreER^* (Harfe et al., 2004) and *Tagln^Cre^* mice (Holtwick et al., 2002) were group housed and genotyped as previously published (sequences are provided in Table S1). Apart from *K14^Cre^*, all mice were acquired from the Jackson Laboratory (JAX) and maintained on a C57BL/6J background. *K14^Cre^* was on a mixed background at the time of acquisition but subsequently crossed to *R26^mT/mG^* (of C57BL/6J background) for more than six generations. The resulting *K14^Cre^*;*R26^mT/mG^* mice were used to produce embryos for the scRNA-seq, RNA *in situ* hybridization mapping and explant culture experiments in this study. For lineage tracing, *Shh^CreER^* and *Tagln^Cre^* were crossed to *R26^mT/mG^* to produce *Shh^CreER^*;*R26^mT/mG^* and *Tagln^Cre^*;*R26^mT/mG^*, respectively. Timed pregnancy was set up either in the morning or in the afternoon to obtain embryos at different stages, as indicated in the text. Noon of the day of vaginal plug discovery was designated as E0.0 or E0.5, depending on the time of breeding setup. Both male and female embryos were selected randomly and used in all experiments. To activate CreER, tamoxifen dissolved in corn oil at a dose of 3.75 mg/30 g body weight was delivered to pregnant *Shh^CreER^*;*R26^mT/mG^* females at E9.5 or *Tagln^Cre^*;*R26^mT/mG^* females at E11.5 through oral gavage. For 5-ethynyl-2′-deoxyuridine (EdU) incorporation, 100 μl of EdU (10 mg/ml, Thermo Scientific) was given to pregnant females through intraperitoneal injection 30 min before sacrificing. All mice were maintained in the University of California Los Angeles (UCLA) pathogen-free animal facility. All experiments involving mice were approved by the Institutional Animal Care and Use Committee of UCLA (Protocol Number ARC-2019-013).

### Single cell isolation from mouse embryonic mandibles

The protocol for single cell dissociation was modified from previous studies (Haber et al., 2017; Sharir et al., 2019). Mandibles from E12.0 and E9.5 mouse embryos were used for single cell isolation in this study. To label the epithelium at E12.0 for easier downstream processing, we used *K14^Cre^*;*R26^mT/mG^* embryos, where Keratin 14-driven Cre recombinase permanently labels epithelial cells with membrane GFP (mG) from the *R26^mT/mG^* Cre reporter beginning at E11.0. Mesenchymal cells lack Cre activity and continue to express membrane tdTomato (mT). To isolate E12.0 mandibular epithelial cells for scRNA-seq, we harvested six E12.0 *K14^Cre^*;*R26^mT/mG^* mouse embryos from the same litter and dissected out their mandibles in cold HBSS (Gibco) (Fig. 1A). Mandibles were then pooled and incubated with 10 mg/ml Dispase II (Sigma-Aldrich) in HBSS supplemented with 10 µg/ml DNase (New England Biolabs) at 37°C and swirled at 100 rpm for 32 min to enzymatically separate the epithelium from the mesenchyme, as previously described (Li et al., 2016b) (Fig. 1B). This allows a more uniform dissociation process and capture of most epithelial cells, which are more adhesive than the mesenchyme and comparatively far fewer in number at this stage. After peeling off the epithelia using forceps, they were dissociated in TrypLE (Gibco) at 37°C for 30 min, with gentle pipetting every 10 min. Cells were then centrifuged at 400 ***g*** for 5 min and resuspended in cold flow cytometry buffer [calcium free HBSS with 5% fetal bovine serum (FBS, Gibco), 2 mM EDTA, and 10 mM HEPES (Gibco)]. Undissociated cell clumps were sieved out using a 20 µm pluriStrainer. As cell doublets can confound scRNA-seq data interpretation (Stegle et al., 2015), the resulting cell suspension was sorted using fluorescence-activated cell sorting (FACS) to isolate GFP^+^ single epithelial cells (Fig. 1C).

To collect E9.5 mandibular cells, mandibular arches from eight E9.5 *K14^Cre^*;*R26^mT/mG^* mouse embryos in the same litter were pooled and directly dissociated in TrypLE at 37°C for 19 min, with gentle pipetting at 12 min. Cells were collected by centrifugation at 250 ***g*** for 4 min. As E9.5 cells were easily dissociated into single cell suspension, FACS was not performed. Cell numbers and viability were analyzed for both E12.0 and E9.5 dissociated cells using the Invitrogen Countess II FL, which showed greater than 90% viability in both samples.

### Single cell RNA-seq: barcoding, library construction and data analysis

The live single cell numbers in suspension were adjusted to a final concentration of 1000 cells/µl in PBS with 0.04% BSA and ∼14,000 cells were loaded to a 10X Chromium Single Cell instrument for single cell partitioning at the UCLA Technology Center for Genomics and Bioinformatics (TCGB). Sample barcoding, cDNA amplification and library construction were performed using the Chromium Single Cell 3′ Library Kit v3 according to the manufacturer's instructions. The two cDNA libraries, one from E12.0 and one from E9.5 mandibles, were confirmed for their qualities using an Agilent TapeStation. A total of 11,131 cells from E12.0 mandibles and 13,081 cells from E9.5 mandibles were successfully sequenced on an Illumina NextSeq 500 system, which produced about 218 million and 568 million reads, respectively.

The sequencing reads were aligned against GRCm38 using CellRanger 2.0.0. Further downstream analyses were conducted in R (version 4.1.2) using the R package Seurat (version 4.0) (Hao et al., 2021). Following the standard Seurat workflow, we first filtered out low-quality cells with less than 200 or over 5500 unique feature counts or with more than 10% mitochondrial gene counts. The filtered dataset was then normalized using Seurat's SCTransform function (Hafemeister and Satija, 2019). To reduce the effects of cell cycle and sex heterogeneity in our scRNA-seq data, we first used Seurat's CellCycleScoring or AddModuleScore functions to assign scores to these categories based on a list of cell cycle genes (G2/M and S markers) (Tirosh et al., 2016) and sexually dimorphic genes (*Uty*, *Ddx3y*, *Kdm5d*, *Eif2s3y*, *Xist*, *Tsix* and *Lars2*) (Huang et al., 2021; Lowe et al., 2015), which were then regressed out from the count matrix as described in the Seurat documentation. Using AddModuleScore, we have also regressed out genes encoding lincRNAs, Gm42418 and AY036118, which overlap with the rRNA Rn45s locus and can be differentially amplified as an artefact at the amplification step (Kimmel et al., 2020).

We next performed dimensionality reduction by principal component analysis (PCA) and uniform manifold approximation and projection (UMAP), followed by unsupervised cell clustering using the FindNeighbors and FindClusters functions. The number of top principal components (PCs) used for dimensional reduction and the resolution of clustering were guided by Seurat's ElbowPlot and the Clustree package (Zappia and Oshlack, 2018), respectively. For the E12.0 data, this allowed us to first obtain an overview of the general cell populations using a low-resolution parameter (10 PCs and 0.08 resolution) and then examine the constituent sub-populations in greater detail with a high-resolution setting (30 PCs and 0.9 resolution). For the E12.0 peridermal and tongue epithelium clusters, we further subset these cells and iterated clustering to identify the different cell types within. To analyze the data from E9.5 mandibles, we first subset the epithelial populations (3944 cells) from the mesenchymal and endodermal cells, and then used 30 PCs and 0.2 resolution for clustering. The E10.5 mandibular epithelial scRNA-seq data were subset from a previously published whole-mandible single cell dataset (Xu et al., 2019), pre-processed with SCTransform and regression of effects from cell cycle, sex and lincRNAs, and then integrated into our E12.0 dataset using Seurat, as previously described (Stuart et al., 2019).

Differentially expressed marker genes for each cluster were identified by Seurat's FindAllMarkers function using the Wilcoxon Rank Sum test, with the cutoff criteria set for genes expressed in a minimum of 15% of cells and a fold change of 1.3 (Table S2). Functional enrichment analysis for top ranked cluster(s)-specific marker genes with an adjusted *P*-value *P*_val_adj≤1×10^−50^ was performed using Metascape (http://metascape.org) (Zhou et al., 2019) (Table S3). The functional enrichment *P*-values were generated by Metascape using cumulative hypergeometric distributions and the default cutoff *P*-value 10^−2^ was used. Top ranked GO terms that better inform signaling pathways or cell biological functions and processes in the context of epithelial development are selected for presentation in figures. As most marker genes in clusters Di are not highly specific and are also enriched in neighboring clusters, we did not include its functional enrichment analysis in this study. For clusters with similar expression profiles and gene functions, their marker genes were merged and collapsed as a single input for the Metascape analysis. Last, differentially expressed genes with a pct. 2<0.5 (i.e. detected in less than 50% of the cells in other clusters) were used to assess the number of markers co-expressed by cells in the dental, taste bud and salivary gland clusters.

To explore the potential epithelial lineages in the developing mandible, we performed RNA velocity using scVelo (version 0.2.4) (Bergen et al., 2020) with *n*_top_genes=2000, *n*_pcs=30 and *n*_neighbors=30 under the dynamic mode, and then projected the velocity field onto Seurat-generated UMAPs. To identify differentially expressed key transcription factors and their downstream gene regulatory networks, we applied SCENIC (single cell regulatory network inference and clustering) to analyze our data using the default setting (Aibar et al., 2017). To better visualize the network, we also examined marker genes from each or combined clusters using the iRegulon plug-in from Cytoscape (Janky et al., 2014), setting the putative regulatory region at 500 bp upstream and an enrichment score threshold of 2.5. Finally, CellChat (Jin et al., 2021) was used to infer major cell-cell singling networks in the E12.0 epithelial population and to identify contributing ligand-receptor pairs.

### RNA *in situ* hybridization

Mouse embryos at different stages were dissected out from the uterus in DEPC-treated PBS. For whole-mount RNA *in situ* hybridization (WISH), the mandibles were collected and fixed with 4% paraformaldehyde (PFA) in DEPC-treated PBS overnight at 4°C. WISH was carried out as previously described (Hu et al., 2012). For each marker gene, we designed two antisense digoxigenin-labeled probes whenever possible, unless restricted by gene size, sequence homology or cloning challenges (Table S1). Hybridized tissues were detected by BM Purple (Roche) and imaged using a Leica DFC7000T camera fitted on a Leica M205 stereomicroscope. To further analyze gene expression in the epithelium at a finer resolution, the stained whole-mount samples were processed through serial sucrose washes and embedded in the Tissue-Tek OCT compound (Sakura Finetek) for frozen sections. Sections (10 μm) were obtained using a Leica CM3050S Cryostat and imaged using a Leica DM 1000 microscope. For samples with weak signals following sectioning, we instead performed section RNA *in situ* hybridization on paraffin sections (7 μm) using established protocols (Hu et al., 2017).

For RNAscope analysis, embryonic heads were dissected and fixed in 10% neutral buffered formalin for 24 h at room temperature and dehydrated through serial ethanol washes, embedded in paraffin wax and sectioned at 6 μm. RNAscope was carried out using the RNAscope Multiplex Fluorescent v2 Assay (Advanced Cell Diagnostics) by following the manufacturer's instructions. Optimized tissue pretreatment steps include boiling sections in the Target Retrieval Reagents (Advanced Cell Diagnostics) at 100°C for 10 min and incubating samples in the Protease Plus solution (Advanced Cell Diagnostics) at 40°C for 10 min. Opal 520, 570 and 690 from Akoya Biosciences were used for color development. A RNAscope 3-plex Negative Control Probe (Advanced Cell Diagnostics) consistently showed no background staining. RNAscope *Mus musculus* probes for *Bdnf*, *Cxcl14*, *Ddit4l*, *Dmrt2*, *Dsc3*, *Irx1*, *Ntrk2*, *Pitx2*, *Prss23*, *Rxfp1*, *Shh*, *Sox2*, *Tfap2b*, *Vgll2*, *Rtl3* and *Zfp750* were purchased from Advanced Cell Diagnostics. The *Pitx2* probe set recognizes all isoforms that are shown to have similar expression patterns at the stages examined in this study (Liu et al., 2003).

### Immunofluorescence staining

For immunofluorescence staining, samples were fixed in 4% PFA overnight at 4°C and prepared as either frozen or paraffin wax-embedded sections. For paraffin sections, antigen retrieval was performed by sub-boiling slides in a microwave for 15 min in citric buffer (pH 6.2) containing 10 mM citric acid, 2 mM EDTA and 0.05% Tween-20. After blocking tissues with a blocking solution [1× animal-free blocker (Vector Laboratories), 2% heat inactivated goat serum, 0.02% SDS and 0.1% Triton-X] for 1 h, slides were incubated with the primary antibodies against the following proteins overnight at 4°C: ACTA2 (Abcam, ab8211), E-cadherin (Cell Signaling, 3195S), GFP (Abcam, ab13970), laminin (Sigma-Aldrich, L9393) and p21 (BD Biosciences, 556430). All antibodies were diluted at 1:100 in the same block without serum. Secondary antibodies (Thermo Scientific) used were Alexa Fluor 555 (A32732) and Alexa Fluor 488 (A32731) goat anti-rabbit IgG, Alexa Fluor 488 goat anti-mouse IgG (A32723), and Alexa Fluor 488 goat anti-chick IgG (A11039), all at 1:250 dilution for 1 h at room temperature. p21 was detected first by a biotinylated anti-mouse secondary antibody, and then sequentially amplified using VECTASTAIN Elite ABC HRP Kit (Vector Laboratories) and Tyramide Signal Amplification (PerkinElmer). DAPI (Invitrogen) was used as a nuclear stain. EdU labeling was detected using a Click-iT Plus EdU Alexa Fluor 555 Assay Kit (Invitrogen, C10638) before primary antibody incubation. Dual immunofluorescence and RNAscope staining was performed following the manufacturer's instructions (RNAscope Multiplex Fluorescent v2 Assay combined with Immunofluorescence - Integrated Co-Detection Workflow). All images were taken using a Zeiss LSM 780 confocal microscope.

### Explant culture

Dissected E11.5 embryonic mandibles were cultured on top of a 0.4 μm Millicell filter (Millipore) supported by a metal mesh (914 μm mesh opening, Spectrum Labs) at the interface of air and media at 37°C and 5% CO_2_ as previously reported (Du et al., 2017). The culture media contains BGJb medium (Gibco), 3% FBS (Gibco), 1% MEM non-essential amino acids (Gibco), 1% GlutaMax (Gibco), 140 μg/ml L-ascorbic acid (Thermo Scientific), 1% penicillin-streptomycin (Thermo Scientific) and the NTRK2 inhibitor ANA-12 (150 μM, Sigma-Aldrich) or an equal volume of DMSO control vehicle. The mandible explants were cultured for either 48 or 72 h before processing for paraffin sections. For labeling cycling cells with EdU, 5 μl of EdU (10 mg/ml, Thermo Scientific) was directly pipetted on top of the explants and then incubated for 4 h before processing tissues for frozen sections. The 4 h pulse time was optimized to ensure sufficient labeling, as shorter pulses marked fewer cells due to slower explant development. Quantification of the tooth germ size was carried out using ImageJ (version v1.53q).

### Statistics and reproducibility

All experiments, except scRNA-seq and RNA *in situ* hybridization, were replicated at least three times using independent biological samples. Marker genes identified from scRNA-seq were validated by RNA *in situ* hybridization studies, which were conducted in at least two independent biological experiments per probe. RNAscope was replicated in at least three independent biological experiments for each set of double Z oligo probes. All images are representative. Each data point in Figs 7K-M and 9B,D, and Fig. S10F represents a single biological sample. Data points were collected without investigator blinding. No data were excluded. Graphs were prepared using the Prism software and data are mean±s.d. (standard deviation). *P-*values were calculated as specified in figure legends. Significance was taken as *P*<0.05 with a confidence interval of 95% (*P<0.05; **P<0.01; ***P<0.001; ****P<0.0001).

## Supplementary Material

Supplementary information

Reviewer comments
