# Supplementary material for: Understanding the development of oral epithelial organs through single cell transcriptomic analysis
Source: Development. 2022 Aug 17;149(16):dev200539. doi: 10.1242/dev.200539 (PMC9481975; doi:10.1242/dev.200539)
Supplement: Supplementary information [file develop-149-200539-s1.pdf]

Figure S1

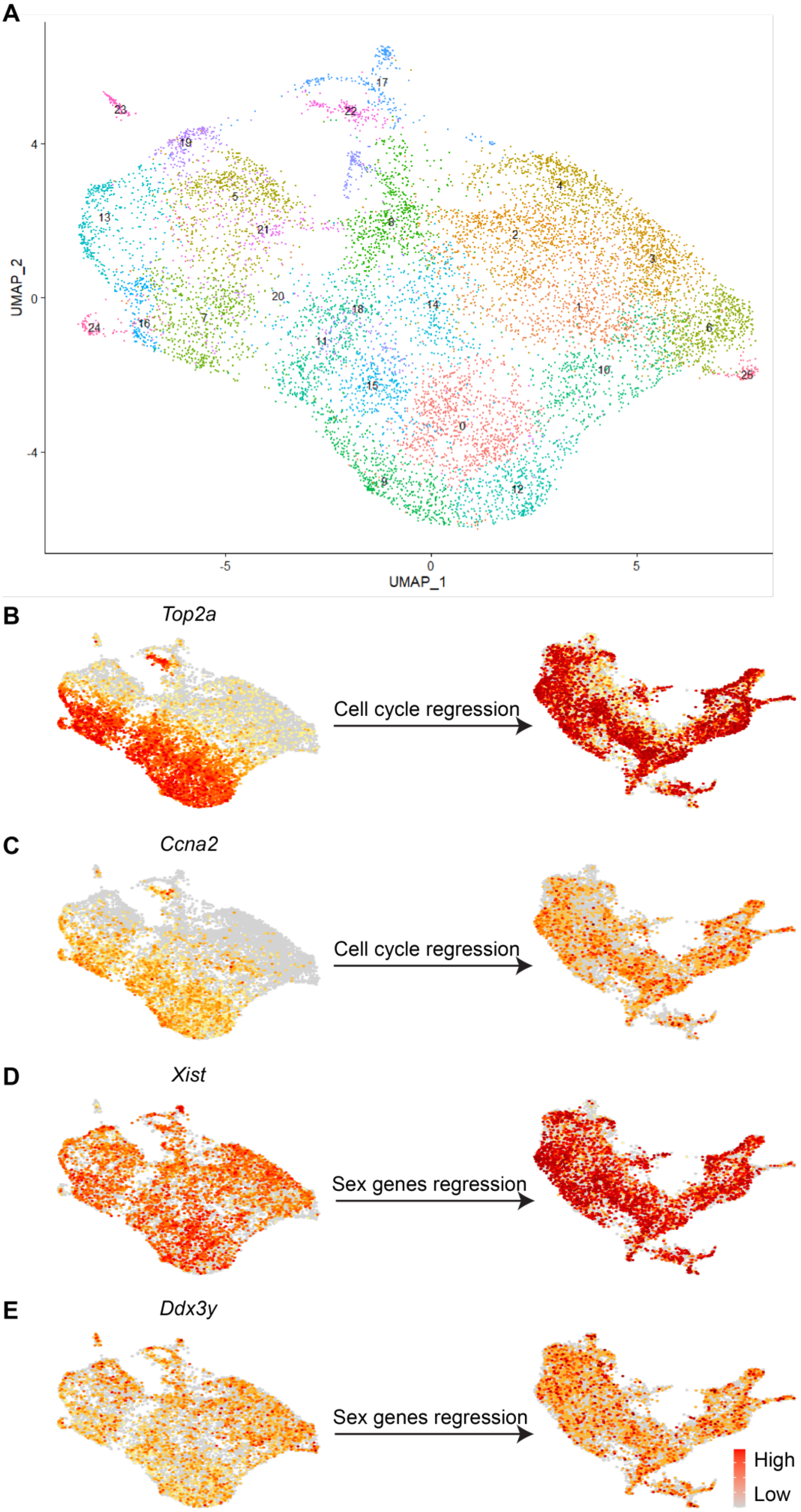

Development • Supplementary information

Fig. S1. UMAP clustering before and after regression of cell cycle and sexually dimorphic genes. (A) UMAP plot showing cell clusters before regressing out cell cycle- and gender-related genes. (B-E) Feature plots showing examples of cell cycle (*Top2a* and *Ccna2*) and sexually dimorphic (*Xist* and *Ddx3y*) genes before and after the regression. Differences in cell cycles contribute greatly to the initial clustering, separating out clusters with otherwise closely matched expression profiles into cycling (bottom half) and non-cycling (top half) populations.

Figure S2

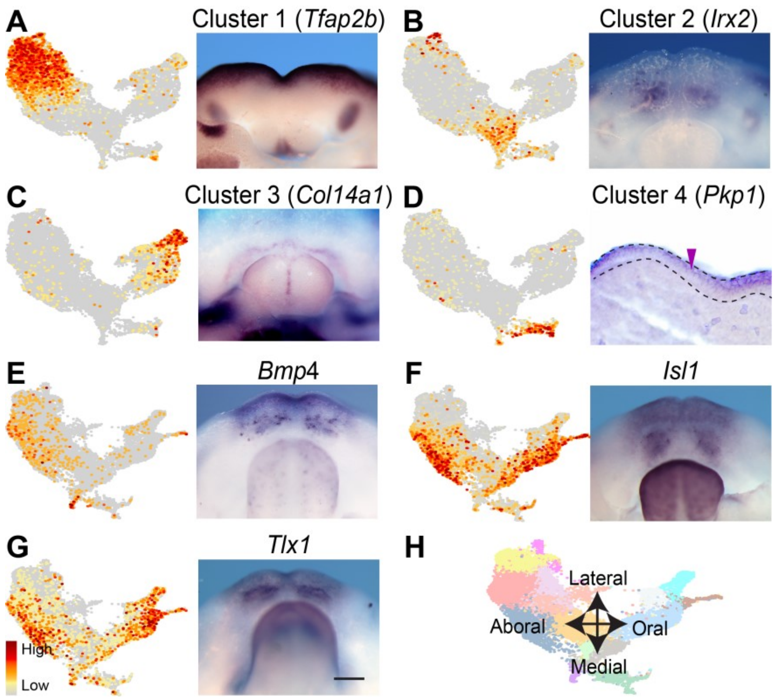

Fig. S2. Expression of marker genes in different patterning domains. (A-C) Expression of *Tfap2b* (cluster 1 marker), *Irx2* (cluster 2 marker), and *Col14a1* (cluster 3 marker) in feature plots and dorsal views of E12.0 whole mount mandibles, labelling the anterior, the dental, and the posterior epithelium respectively.(D) Expression of the cluster 4 marker, *Pkp1*, in its feature plot and in the periderm layer (purple arrowhead) on a E12.0 mandible sagittal section. Dashed lines outline the epithelium. (E-G) Expression of known incisor markers, *Bmp4*, *Isl1*, and *Tlx1*, that label the medial portion of E12.0 mandibles (dorsal views shown). (H) The distribution of clusters on the UMAP corresponds to the oral-aboral and medial-lateral axes of the mandible. Scale bar in (G) represents 280 µm in (A-C,E-G) and 50 µm in (D).

Figure S3

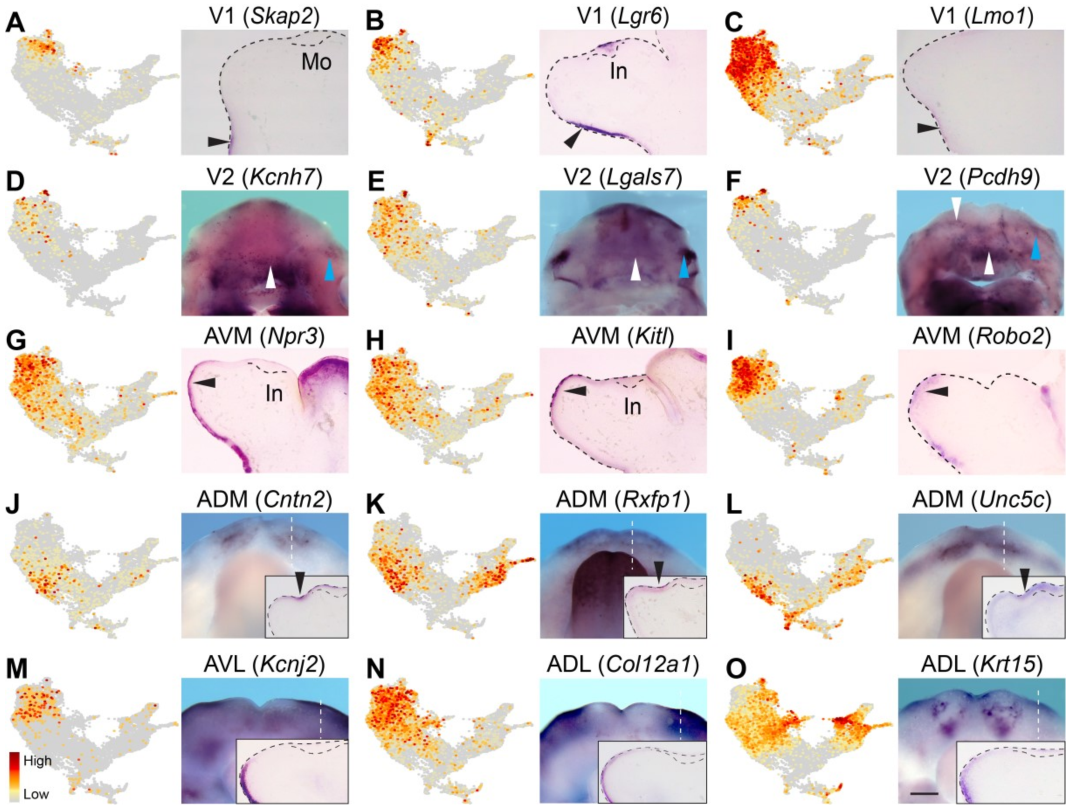

Fig. S3. Mapping the anterior epithelial clusters by RNA *in situ* hybridization. (A-O) Feature plots and *in situ* hybridization of indicated marker genes for clusters V1 (ventral 1), V2 (ventral 2), AVM (anteroventral-medial), ADM (anterodorsal-medial), AVL (anteroventral-lateral), and ADL (anterodorsal-lateral) on E12.0 mouse mandibles. Sagittal sections are shown in (A-C,G-I) and in the insets of (J-O); anterior to the left. Whole mount mandibles are shown in ventral views (D-F) and dorsal views (J-O). Black dashed lines in sections outline the mandible and the dental epithelium. Black arrowheads indicate expression of marker genes that help assign cluster identities. White and cyan arrowheads in (D-F) mark the respective puncta and lateral patch expression pattern of V2 markers. In, incisor; Mo, Molar. Scale bar in (O) represents 100 μm in (A-C,G-I), 450 μm in (D-F,M), 280 μm in the whole mount images in (J-L,N,O), and 200 μm in the insets in (J-O).

Figure S4

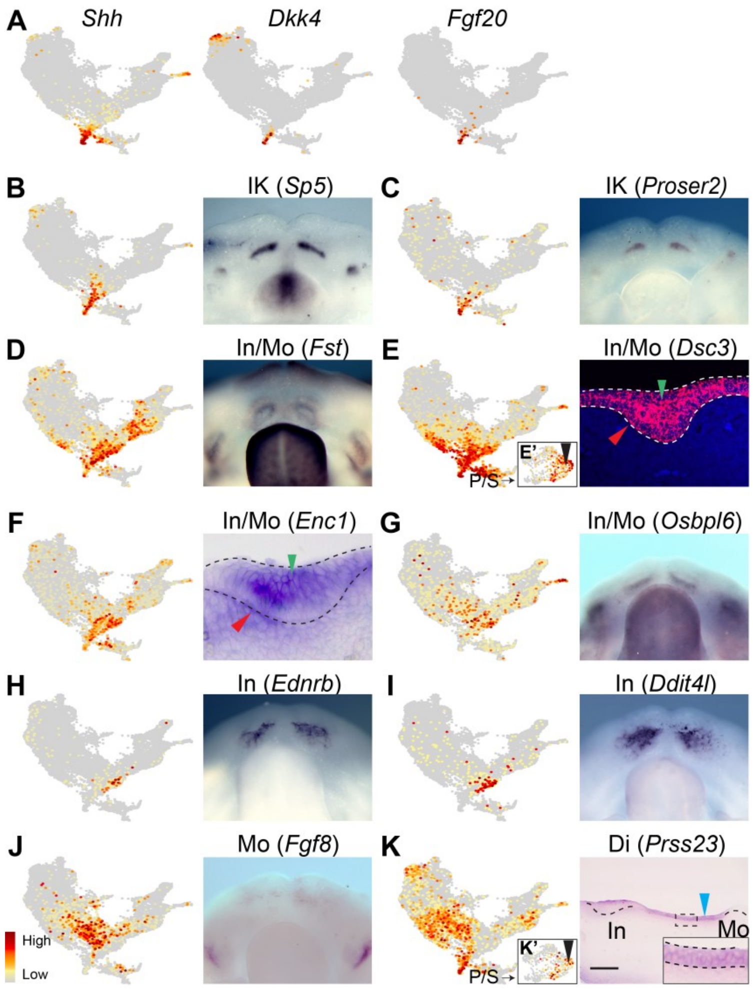

Fig. S4. Mapping tooth-related clusters. (A) Feature plots for known initiation knot markers *Shh*, *Dkk4*, and *Fgf20* . (B-J) Feature plots and *in situ* hybridization of selected marker genes for clusters IK, In, and Mo, on E12.0 mouse mandibles. Whole mount mandible in dorsal views are shown in (B-D,G-J). (E,F) *Dsc3* and *Enc1* are expressed in the entire incisor epithelium, including the initiation knot (red arrowheads) and all suprabasal cells (green arrowheads), as shown on sagittal sections using RNAscope (E) or section *in situ* hybridization (F); anterior to the left. (K) *Prss23* is expressed in the diastema region (cyan arrowhead), as shown on a diagonal section through both the incisor and the molar. The enlarged image of the dashed box is shown in the inset. (E',K') Subclustering of the P/S cluster shows that *Dsc3* and *Prss23* are enriched in subcluster P/S2 (black arrowheads). Di, diastema; IK, initiation knot; In, incisor; Mo, Molar. Scale bar in (K) represents 280 μm in (B-D,G-J), 70 μm in (E-F), 100 μm in (K), and 25 μm in the inset in (K).

Development • Supplementary information

Figure S5

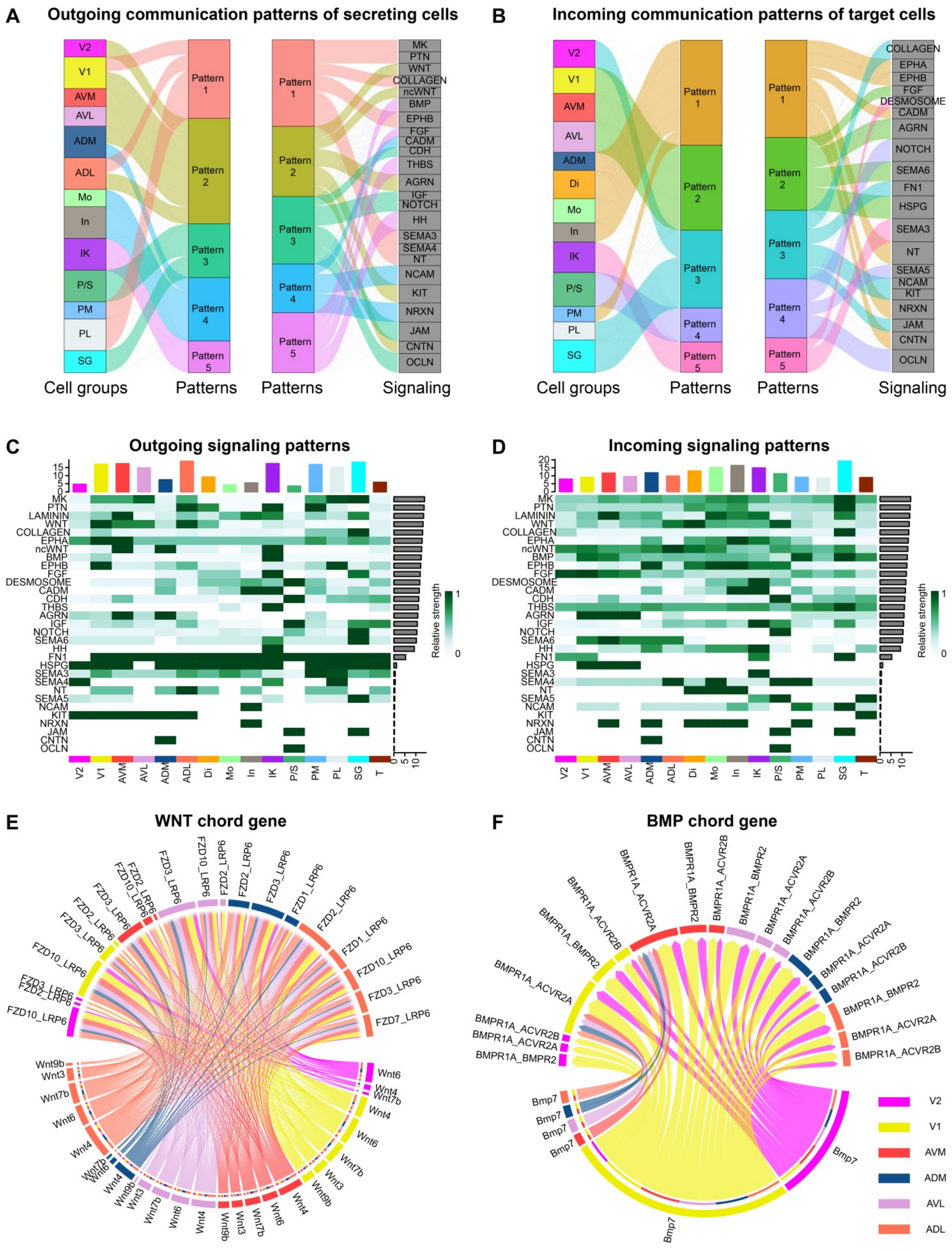

Development • Supplementary information

Fig. S5. CellChat inference of major outgoing and incoming signaling pathways in the developing mandibular epithelium at E12.0. (A,B) River plots showing the outgoing and incoming signaling patterns of secreting (A) and target (B) populations. The thickness of the flow represents the relative contribution of the cell group or signaling pathway to each pattern. (C,D) Heatmap showing the dominant outgoing (C) and incoming (D) signals in each cell group. (E,F) Chord diagrams showing possible WNT (E) and BMP (F) ligand-receptor interactions within and between anterior epithelial populations.

Figure S6

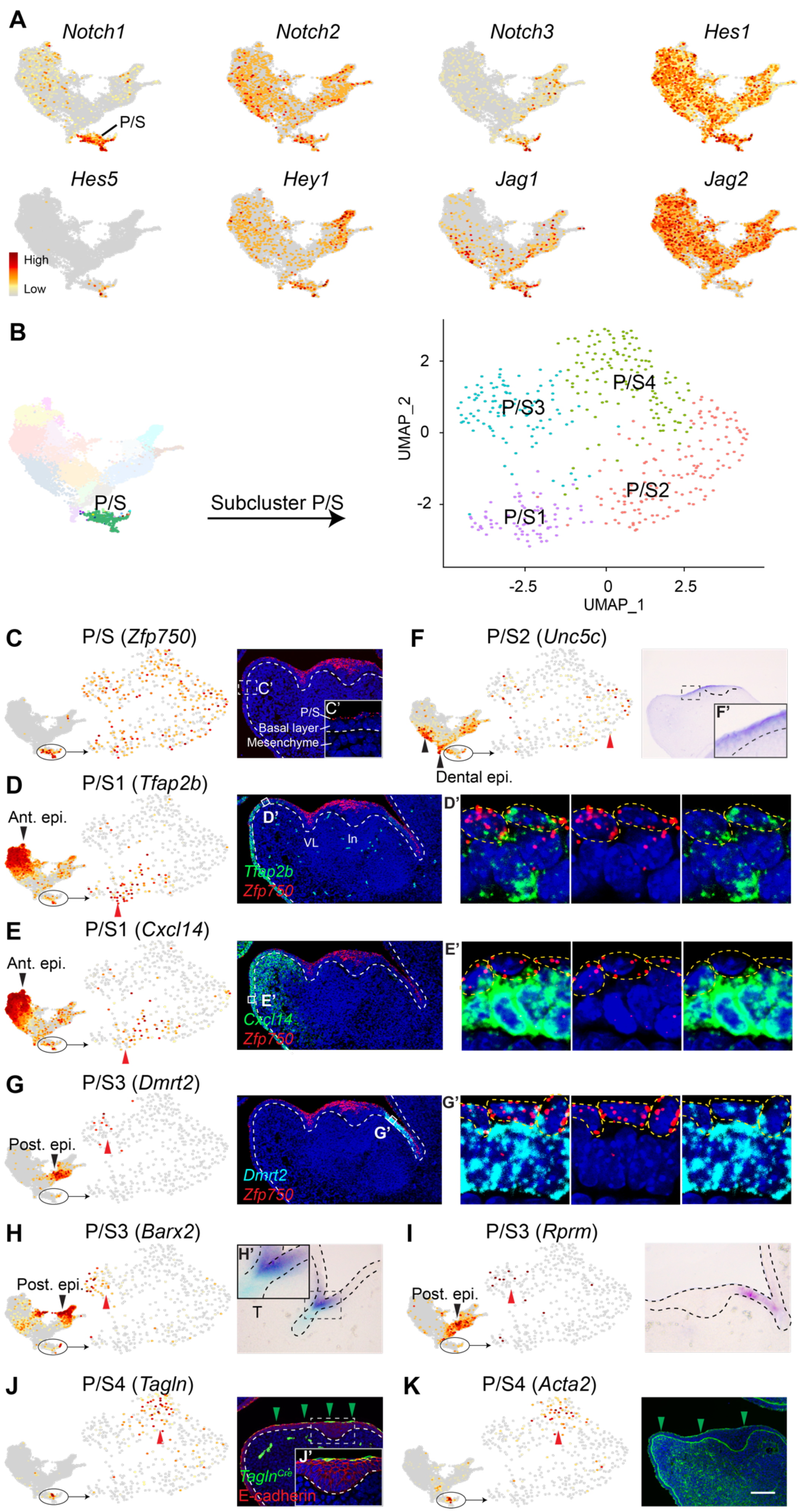

Development • Supplementary information

Fig. S6. Subclustering the periderm and suprabasal (P/S) cluster. (A) Feature plots for genes encoding components of the Notch signaling pathway. Notch receptors and Notch responsive genes *Hes1*, *Hes5*, and *Hey1* are expressed in cluster P/S. (B) Cluster P/S is subclustered into P/S1-4. (C-K) Feature plots of indicated markers for P/S1-4 and their expression pattern, as assessed using RNAscope (C-E,G), section *in situ* hybridization (F,H,I), $\textit{Tagln}^{\textit{Cre}}$ (J), and immunostaining (K). Sagittal sections through the incisor bud are shown in (C-G,I-K); anterior to the left. Frontal section is shown in (H). (C'-H',J') are enlargements of corresponding boxes. Dashed lines outline the epithelium or the incisor bud. *Zfp750* is a pan-P/S marker and labels both the periderm and the suprabasal cells, but not the basal layer (C). P/S1-3 express the same sets of regional markers as the rest of the epithelium (red and black arrowheads in feature plots), thus corresponding to the periderm and suprabasal cells that retain the positional information of the anterior (ant.), the dental, and the posterior (post.) epithelium (epi.) (D-I). For example, *Zfp750*+ anterior (P/S1) and posterior (P/S3) peridermal cells (yellow dashed lines, D',E',G,) express anterior (*Tfap2b* and *Cxcl14*) or posterior (*Dmrt2*) markers that are also present in the basal cells. P/S4 markers *Tagln* and *Acta2* are expressed in a subgroup of peridermal cells over the epithelial surface (green arrowheads), but not in the rest of the suprabasal cells (J,K). Membrane GFP in (J) is expressed from the Cre reporter $\textit{R26}^{\textit{mT/mG}}$ and the epithelium is visualized using E-cadherin antibody (red). In, incisor; T, tongue; VL, vestibular lamina. Scale bar in (K) represents 100 µm in (C-G,J,K), 50 µm in (H,I,J'), 25 µm in (C',F',H'), and 6 µm in (D',E',G').

Figure S7

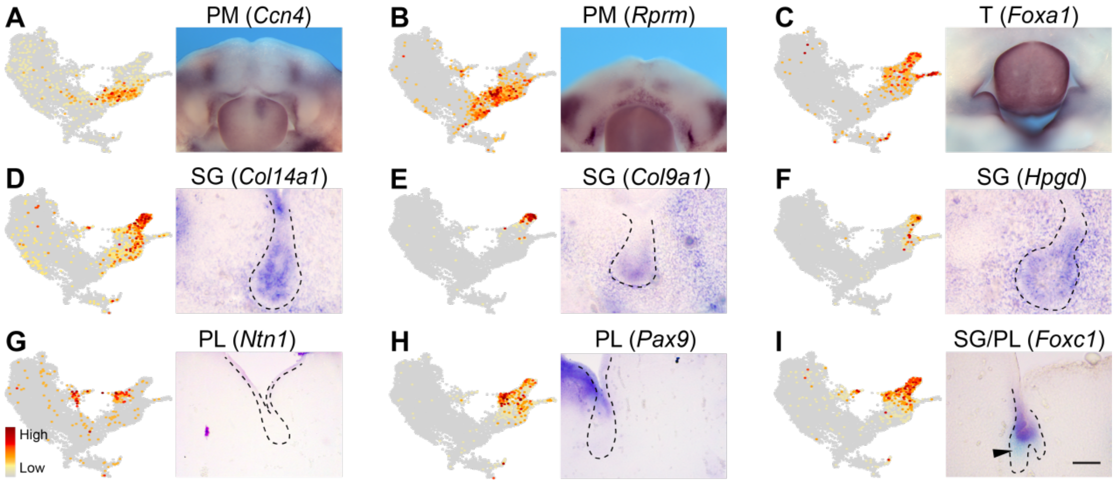

Fig. S7. Identification of the posterior mandibular clusters. (A-C) Expression of PM (posterior-medial) markers *Ccn4* (A) and *Rprm* (B) and cluster T (tongue) marker *Foxa1*, as shown by feature plots and whole mount *in situ* hybridization on E12.0 mandibles (dorsal views). (D-I) Feature plots and frontal sections of the submandibular salivary gland showing the expression pattern of markers for clusters SG (salivary gland) and PL (posterior-lateral). Faint *Foxc1* expression is also detected in the salivary gland (black arrowhead). Dashed lines outline the salivary gland. Scale bar in (I) represents 280 μm in (A-C) and 50 μm in (D-I).

Figure S8

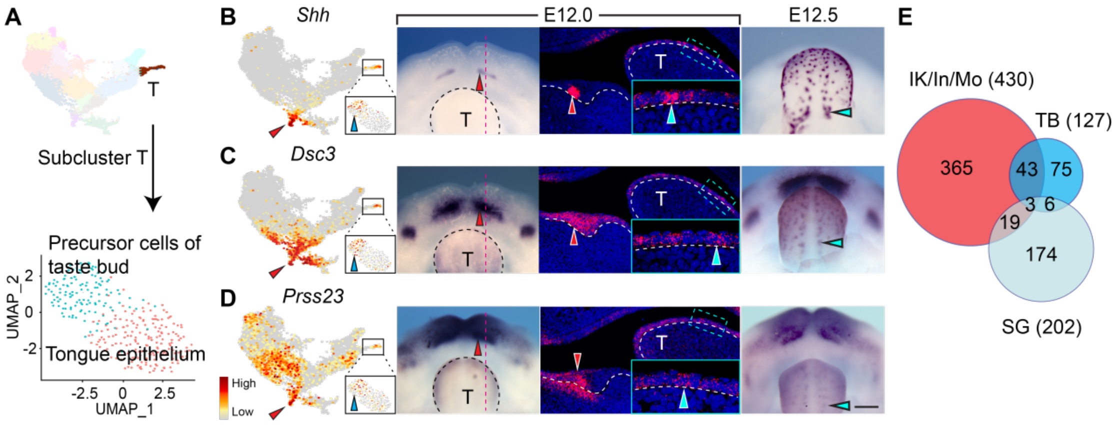

Fig. S8. Identification of taste bud primordia precursor cells in the tongue (T) epithelial cluster. (A) Cluster T is composed of two subclusters, separating the taste bud precursor cells from the rest of the tongue epithelium. (B-D) Feature plots, whole mount RNA *in situ* hybridization (second and fourth panels, dorsal views of E12.0 and E12.5 mandibles), and RNAscope (third panels, sagittal sections at levels indicated by the pink dashed lines) showing *Shh* (B), *Dsc3* (C), and *Prss23* (D) as markers for both the taste bud primordia (cyan arrowheads) and the initiation knot (red arrowheads). White dashed lines outline the epithelium. Black dashed lines outline the tongue. Cyan boxes are enlargements of the tongue epithelium.(E) Venn diagram showing numbers of marker genes shared between the dental (IK/In/Mo), the taste bud (TB), and the salivary gland (SG) clusters. Scale bar in (D) represents 280 µm in whole mount images, 100 µm in RNAscope images, and 30 µm in the insets.

Figure S9

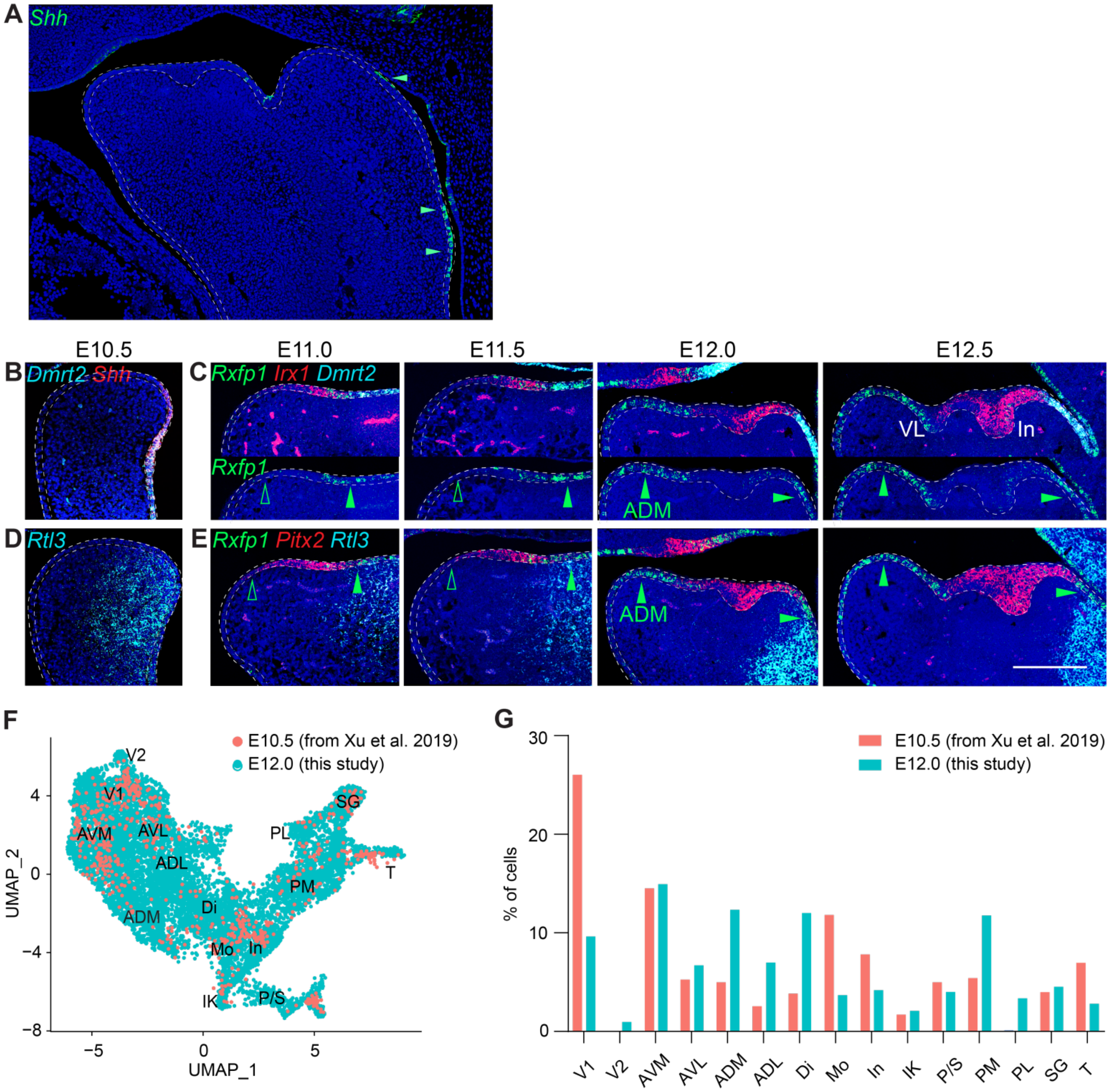

Fig. S9. Changes in the expression of regional markers and the mandibular epithelial populations from E10.5 to E12.0. (A) Sagittal section through the incisor epithelium of a E12.0 mouse mandible showing lineage traced *Shh*-expressing endodermal cells that are genetically labelled with membrane GFP (mG)(green arrowheads) at E9.5 using $Shh^{CreER};R26^{mT/mG}$. (B-E) RNAscope *in situ* staining on sagittal sections through the incisor region (anterior to the left) showing the expression of endodermal marker *Shh* at E10.5, and markers for the dental epithelium (*Irx1* and *Pitx2*), the posterior epithelium (*Dmrt2* and *Rtl3*), and the anterodorsal-medial (cluster ADM) cells (*Rxfp1*) at different stages. (B,D) *Dmrt2* and *Rtl3* are expressed in the endoderm at E10.5 but absent in theectoderm.

Development • Supplementary information

(C,E) *Rxfp1* is a dual ADM and posterior epithelium marker. Open and solid green arrowheads indicate absence or presence of *Rxfp1* expression in the epithelium, demonstrating that ADM formation begins at E12.0. Dashed lines outline the mandibular epithelium. (F) UMAP plot with integrated data from this study (E12.0) and from Xu et al. 2019 (E10.5) showing changes in the composition of epithelial populations. (G) Bar graph showing the percentage of each epithelial population at E10.5 and E12.0. In, incisor; VL, vestibular lamina. Scale bar in (E) represents 200 μm in (A-E).

Figure S10

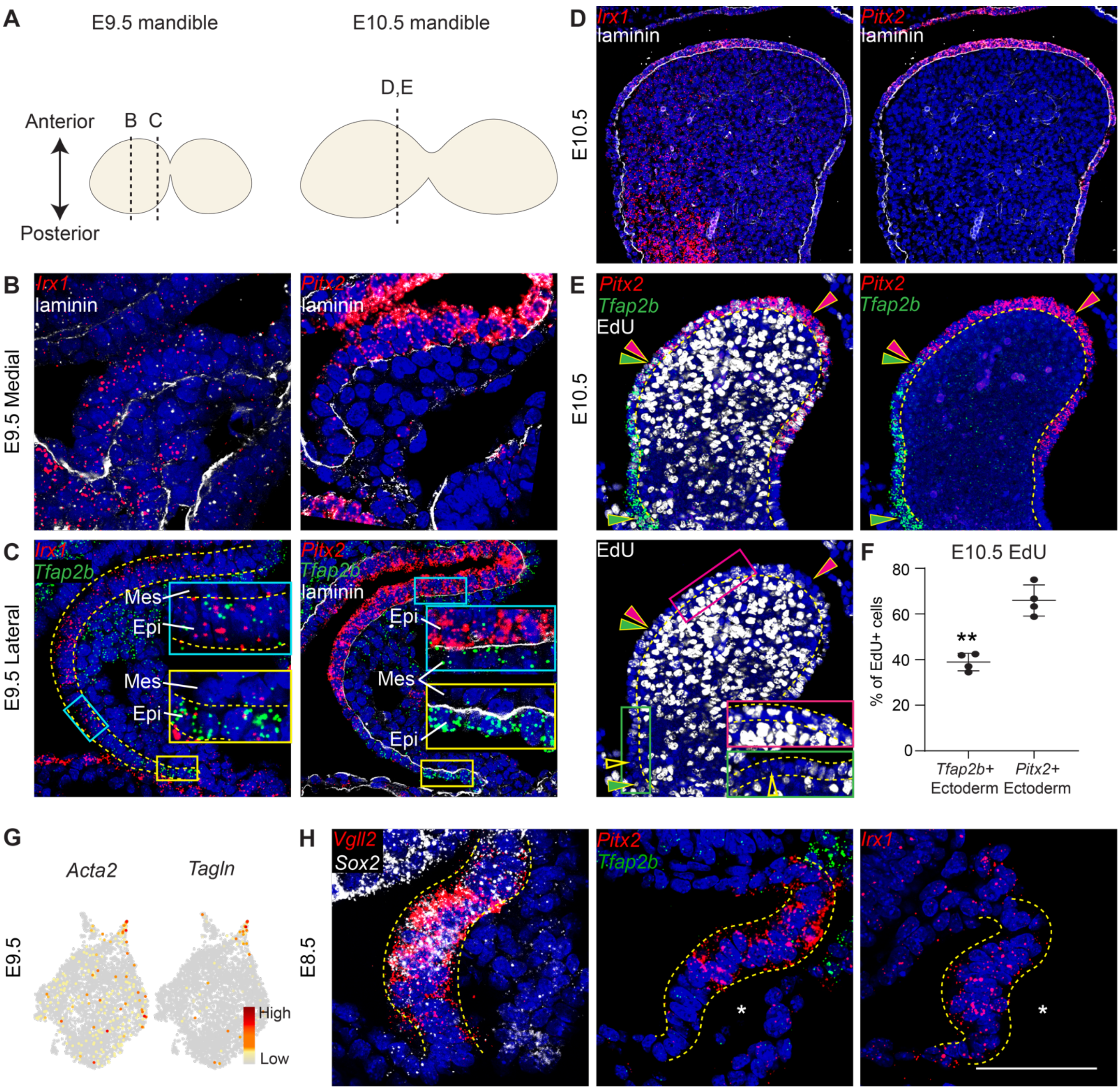

Fig. S10. The expression of oral-aboral regional markers during early mandible development. (A) Schematics of E9.5 and E10.5 mandibles showing levels of medial or lateral sagittal sections. (B-D) RNAscope analysis of *Irx1*, *Pitx2,* and *Tfap2b* expression in E9.5 and E10.5 mandibles. Laminin immunostaining labels the basement membrane and separates the epithelium from the mesenchyme. Cyan and yellow insets show enlarged images of the epithelium. (E,F) Distribution and quantification of EdU+ cells in the *Tfap2b*+ and *Pitx2*+ ectoderm (n=4). Green and pink arrowheads respectively bracket the representative *Tfap2b*+ and *Pitx2*+ ectodermal regions used for the quantification. Green and pink insets

Development • Supplementary information

show enlarged images of the epithelium. Open yellow arrowheads show reduced proliferation in the ventral aboral epithelium (G) Feature plots of indicated markers for mandibular epithelial clusters at E9.5. (H) Expression of *Vgll2*, *Sox2*, *Pitx2*, *Tfap2b*, and *Irx1* by RNAscope in the newly formed mandibular arch epithelium at E8.5. * indicates space occupied by the arch mesenchyme that is lost during sectioning. Yellow dashed lines outline the ectodermal layer. Epi, epithelium; Mes, mesenchyme. Scale bar in (H) represents 60 µm in (B,C,H), 200 µm in (D,E), 20 µm in boxes of (C), and 133 µm in boxes of (E). Quantitative data are presented as mean ± SD. *P*-values are determined using Student's t-test.

Figure S11

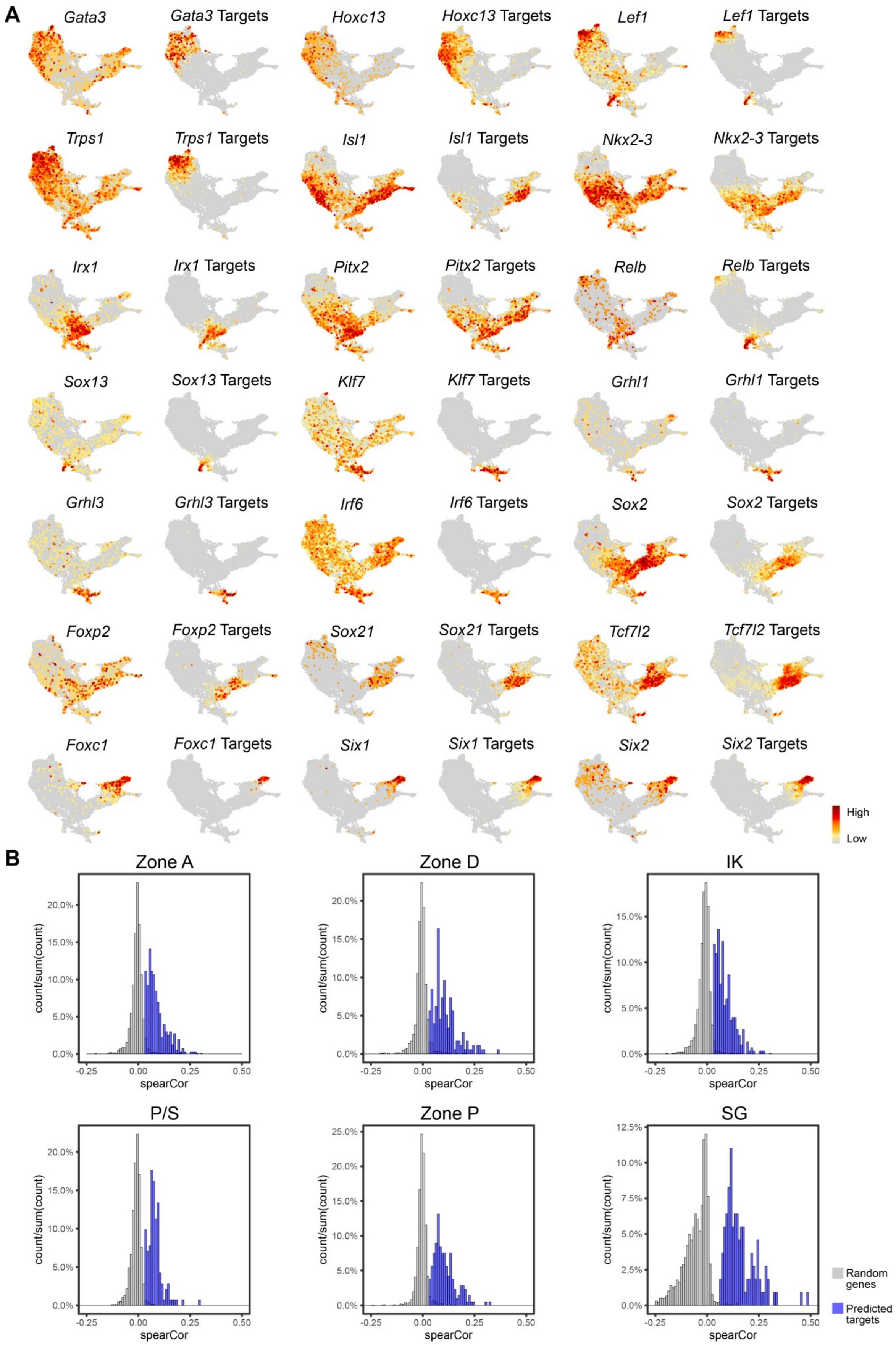

Development • Supplementary information

Fig. S11. SCENIC-identified regulons contain regional and cluster-defining transcription factors and targets that share similar expression patterns in the E12.0 mandibular epithelium. (A) Feature plots showing similar expression patterns of each transcriptional factor and its target genes, calculated as averaged expression in module scores. (B) Histograms showing the expression correlations (Spearman) between all SCENIC-identified targets and their transcription factors (blue bars) and the expression correlations between randomly selected genes and the regional transcription factors averaged in module scores (gray bars).

Development • Supplementary information

Figure S12

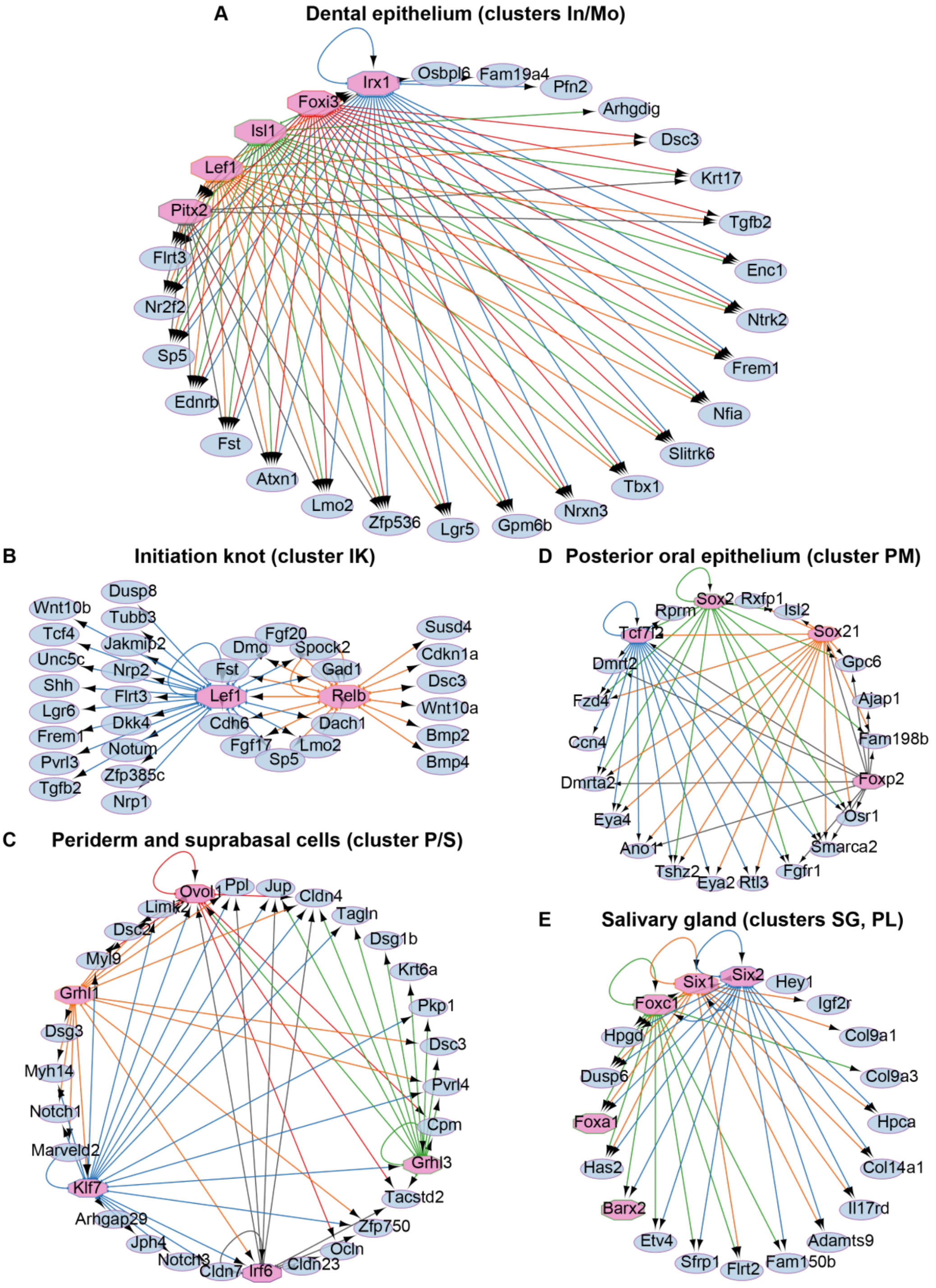

Fig. S12. iRegulon reveals gene regulatory networks in different epithelial populations. (A-E) Gene regulatory networks in the dental epithelium (A), the initiation knot (B), the periderm and suprabasal cells (C), the posterior oral epithelium (D), and the salivary gland (E), as predicted by the iRegulon plugin from Cytoscape. Key transcription factors are labelled in pink and connected to their predicted downstream targets by arrows. All genes shown are enriched in the clusters indicated.

Figure S13

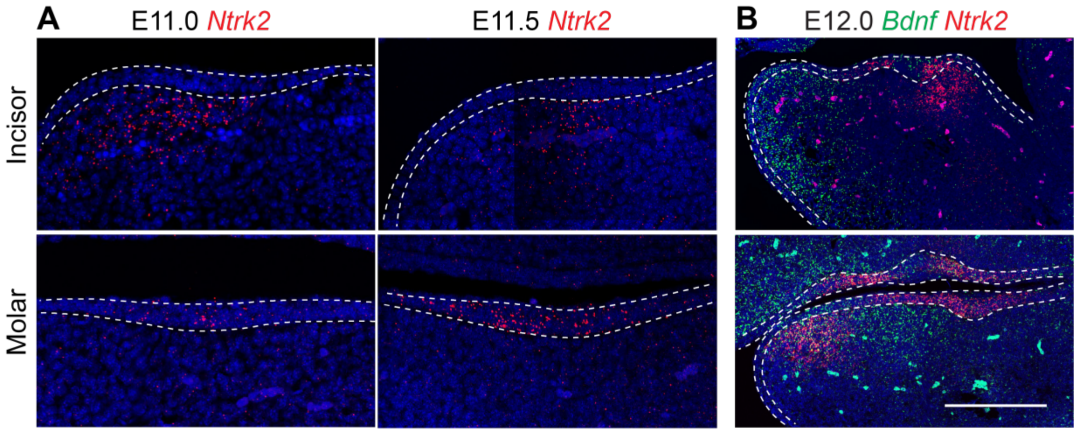

Fig. S13. Expression of *Ntrk2* and *Bdnf*. (A,B) RNAscope *in situ* showing the expression pattern of *Ntrk2* in the incisor and the molar at E11.0-E12.0 and the expression of *Bdnf* at E12.0. Sagittal sections through the incisor region are shown; anterior to the left. Dashed lines outline the epithelium. Scale bar in (B) represents 100 μm in (A) and 200 μm in (B).

Figure S14

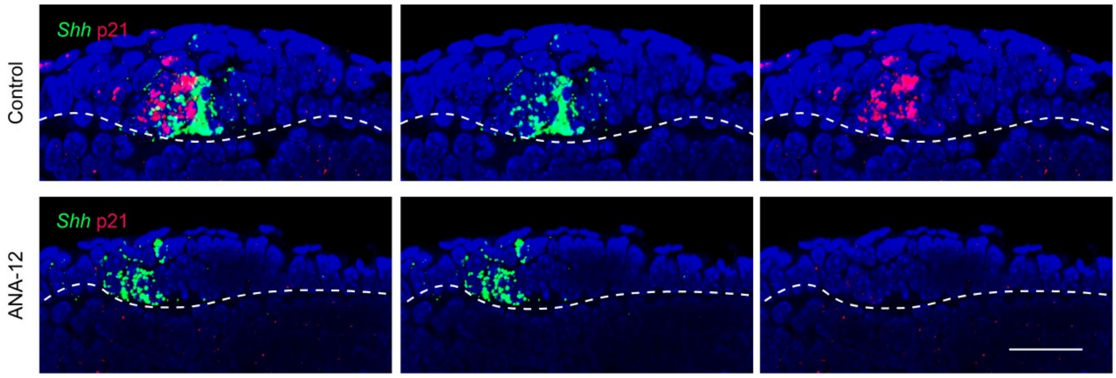

Fig. S14. NTRK2 is required for proper initiation knot maturation. Expressions of the initiation knot marker *Shh* (RNAscope, green) and the cell cycle inhibitor p21 (immunostaining, red) on incisor sagittal sections of E11.5 mandible explants cultured for 48 hours in DMSO (control) or the NTRK2 inhibitor ANA-12; anterior to the left. Dashed lines outline the incisor epithelium. Scale bar represents 30 μm.

Development • Supplementary information

Figure S15

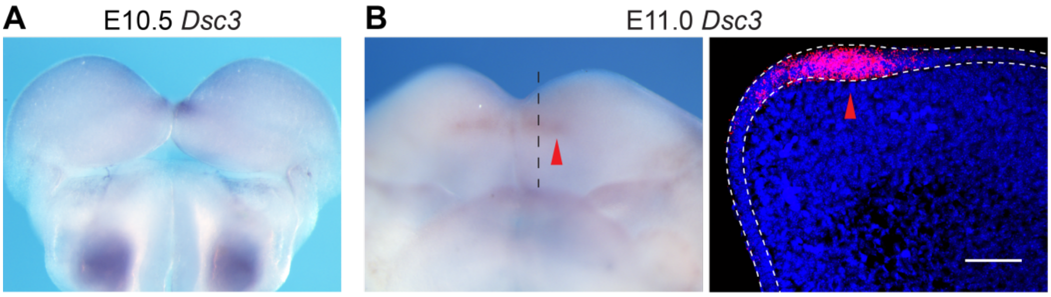

Fig. S15. Expression of *Dsc3*. (A) Whole mount RNA *in situ* hybridization of *Dsc3* (dorsal view of the mandible) showing *Dsc3* is largely absent in the mandible epithelium at E10.5. (B) Whole mount RNA *in situ* hybridization (left panel) showing *Dsc3* expression in the forming dental epithelium at E11.0. Right panel shows *Dsc3* RNAscope *in situ* staining on a sagittal section through the incisor region (indicated by the black dashed line); anterior to the left. White dashed lines outline the epithelium. Red arrowheads point at where *Dsc3* is expressed. Scale bar in (B) represents 280 µm in the whole-mount images in (A,B) and 70 µm in the RNAscope image in (B).

Development • Supplementary information

Table S1. List of primers used for genotyping and cloning of in situ probes.

Click here to download Table S1

Table S2. Differentially expressed genes in the 15 clusters identified from E12.0 mandibular epithelium. *P*-values (p_val) show the probability that a gene is expressed in the cluster. Adjusted *P*-values (p_val_adj) are calculated using Bonferroni correction based on the total number of genes in the dataset. 'avg_log2FC' is the log2 of the mean expression fold-change between cells in an individual cluster and all other cells. 'pct.1' and 'pct.2' are the percentages of cells where the gene is detected in or outside the cluster respectively. The genes were sorted according to 'p_val'.

Click here to download Table S2

Table S3. Functional enrichment analysis for top ranked cluster(s)-specific marker genes using Metascape.

Click here to download Table S3

Table S4. Differentially expressed genes in the subclustered periderm and suprabasal cells.

Click here to download Table S4

Table S5. Differentially expressed genes in the subclustered taste bud precursor cells.

Click here to download Table S5

Table S6. Differentially expressed transcription factors and predicted targets in the mandibular epithelium as identified using SCENIC. Key transcription factors (TF) are listed in column A and their predicted target marker genes are listed in column B. All the listed regulons have high-confidence annotation (column C). 'nMotifs' (column D) is the number of target motifs in the gene. 'bestMotif' (column E) is the name of the most significantly enriched motif. 'NES' (column F) is the standard enrichment score. 'motifDb' (column G) is the search space upstream of the transcription start site. 'coexModule' (column H) is the co-expression modules. 'spearCor' (column I) is the Spearman correlation between the TF and the potential target. 'CoexWeight' (column J) is the co-expression weight between the TF and the target gene.

Click here to download Table S6
