# Supplementary material for: Understanding the development of oral epithelial organs through single cell transcriptomic analysis
Source: Development. 2022 Aug 17;149(16):dev200539. doi: 10.1242/dev.200539 (PMC9481975; doi:10.1242/dev.200539)
Supplement: Reviewer comments [file develop-149-200539_review_history.pdf]

Understanding the development of oral epithelial organs through single cell transcriptomic analysis

Qianlin Ye, Arshia Bhojwani and Jimmy K. Hu
DOI: 10.1242/dev.200539

Editor: Samantha Morris

Review timeline

Original submission:	20 January 2022
Editorial decision:	28 February 2022
First revision received:	27 May 2022
Accepted:	7 July 2022

Original submission

First decision letter

MS ID#: DEVELOP/2022/200539

MS TITLE: Understanding the development of oral epithelial organs through single cell transcriptomic analysis

AUTHORS: Qianlin Ye, Arshia Bhojwani, and Jimmy K. Hu

I have now received all the referees' reports on the above manuscript, and have reached a decision. The referees' comments are appended below, or you can access them online: please go to BenchPressand click on the 'Manuscripts with Decisions' queue in the Author Area.

As you will see, the referees express considerable interest in your work, but have some significant criticisms and recommend a substantial revision of your manuscript before we can consider publication. If you are able to revise the manuscript along the lines suggested, which may involve further experiments, I will be happy receive a revised version of the manuscript. Your revised paper will be re-reviewed by one or more of the original referees, and acceptance of your manuscript will depend on your addressing satisfactorily the reviewers' major concerns. Please also note that Development will normally permit only one round of major revision.

We are aware that you may be experiencing disruption to the normal running of your lab that make experimental revisions challenging. If it would be helpful, we encourage you to contact us to discuss your revision in greater detail. Please send us a point-by-point response indicating where you are able to address concerns raised (either experimentally or by changes to the text) and where you will not be able to do so within the normal timeframe of a revision. We will then provide further guidance. Please also note that we are happy to extend revision timeframes as necessary.

Please attend to all of the reviewers' comments and ensure that you clearly highlight all changes made in the revised manuscript. Please avoid using 'Tracked changes' in Word files as these are lost in PDF conversion. I should be grateful if you would also provide a point-by-point response detailing how you have dealt with the points raised by the reviewers in the 'Response to Reviewers' box. If you do not agree with any of their criticisms or suggestions please explain clearly why this is so.

© 2022. Published by The Company of Biologists under the terms of the Creative Commons Attribution License (https://creativecommons.org/licenses/by/4.0/).

Reviewer 1

Advance summary and potential significance to field

This manuscript describes scRNA-seq analysis of E12 mouse mandibular epithelia, with which the authors identified and verified the patterns of expression of several domain-specific markers and postulated potential gene regulatory networks patterning the mandibular epithelium along the aboral-oral axis. The authors propose an interesting hypothesis that the mandibular epithelium is initially dental-like, with the anterior and posterior regions subsequently specified to delineate the boundary of the tooth filed and confine the dental lamina to its position. They further proposed that the gradual sharpening of the dental lamina epithelium might be through mutual repression of region/cluster-specific transcription factors. Overall, the scRNA-seq dataset from the E12 mandibular epithelium will be a valuable resource and the hypothesized mechanism for regionalization of the mandibular epithelium is interesting. However, the manuscript is mostly descriptive and the major hypothesis remains to be tested.

Comments for the author

In this manuscript, the authors identified regionalization markers of the developing mouse embryonic mandibular epithelium at E12 using scRNA-seq and verified the patterns of expression of selected markers from E9.5 to E12 by in situ hybridization analysis. The bioinformatic analysis of the E12 mandible scRNA-seq dataset was thorough and well presented. The in situ validation data are of high quality. However, while the data generated several interesting hypotheses regarding mechanisms patterning the mandibular epithelium, the manuscript stops short of testing any of the major hypotheses. The only functional study included in the manuscript involved pharmacological inhibition of Ntrk2 in E11.5 mandibular explant cultures with which the authors showed reduction in dental epithelial proliferation in the incisor region but not the molar region at 48 hours after culture. However, several Ntrk2 mutant mouse lines have been reported and there has been no description of mandibular incisor defect, leaving the in vivo role of Ntrk2 in tooth development in question. Thus, while the E12 mandible scRNA-seq dataset and the data analysis presented in Figures 1-8 provide a valuable resource to the developmental biology community, the lack of substantial functional studies to test some of the main hypotheses raised regarding the mechanisms regulating regionalization of the mandibular epithelium is a major limitation for this manuscript.

Reviewer 2

Advance summary and potential significance to field

This resource paper describes the cell diversity of E12.0 mandibular epithelium using scRNA-seq and maps the identified cell populations using in situ hybridization. These studies have identified discrete populations, both in the developing ectodermal organs and in areas where the epithelium appears morphologically simple and uniform. Furthermore, they looked at the expression of a few oral-aboral marker genes at earlier development stages and hypothesize that mandibular ectoderm is initially more dental-like, but newly patterned anterior and posterior regions gradually expand and confine the dental progenitor field to its eventual position. Through computational analysis, this study identified key transcription factors and associated gene regulatory networks for different cell populations. For example, they tested the function of Ntrk2, a novel dental marker identified in this analysis, in the forming incisor using explant culture and discovered that NTRK2 promotes cell proliferation in the invaginating incisor placode. Altogether, this study provides a detailed atlas for the epithelial cell types in developing mandible which serves as an important resource for generating new hypothesis and further investigation the function of specific genes/pathways during epithelial morphogenesis.

Comments for the author

The scRNAseq work is generally well done and my concerns and comments are:

© 2022. Published by The Company of Biologists under the terms of the Creative Commons Attribution License (https://creativecommons.org/licenses/by/4.0/).

Major
1. Since ectoderm is only a very small portion in tissue sections, higher magnification images accompanied with high-quality nuclear staining images are highly recommended for the RNAscope results to see the ectodermal cell layers such as Fig4B,C, Figure 7, and FigS5 C,D,G.
2. It is very helpful to have schematic drawings throughout the paper. It is recommended to incorporate the P/S and V2 clusters into the schematic drawing if possible.
3. It is interesting that the suprabasal cells are clustered with the periderm. It is not clear what the P/S1 and P/S3 cells are since the marker genes used for these clusters are expressed in the corresponding basal cells. To address this, it is recommended to double label with a marker which is expressed only in P/S1 and P/S3 but not in the corresponding basal cells.
4. What is the Seurat package version? Which R version was used? For Metascape, were any p-value cutoffs used for the function enrichment? Were any other filters used for the enriched terms? What types of GO terms were shown in the results? Were merged or overlapped marker genes used for the analysis of multiple clusters?
5. Ntrk2 inhibitor treatment causes smaller tooth buds than controls.
However, more proliferation was observed in the treated incisor IK region. Was this because the IK was not formed in the treated tissue?
6. A data availability section should be included. It is strongly recommended to deposit not only the raw data but also the analyzed scRNA-seq data, including the R objects, in a public repository.
7. For test bud markers were detected in E12.5 tissues in Fig6 E and FigS7B-E and were not detected at E12.0 samples in FigS7B-E by in situ. However they were detected in the E12.0 scRNA-seq. This discrepancy should be addressed.

Minor
1. The last line on the first page, “The mouse jaw” should be “The mouse lower jaw”.
2. In the Methods, on page 4, at the end of paragraph 2, “We have also regressed out genes encoding lincRNAs, Gm42418 and AY036118….”. It is more appropriate to say “removed” instead of “regressed out” if this was done in the same way as in the reference.
3. What does the “mirrored clusters” in first paragraph of the Results section mean?
4. Figure 2C the label on the left side is off one gene for the dental lamina and P/S. Scube1 is a not a marker for P/S, but for dental lamina.
5. The tab names do not match the table names for Table S3-5.

Reviewer 3

Advance summary and potential significance to field

The manuscript by Ye et al succeeds in delivering a comprehensive single-cell expression atlas of the developing mandibular epithelium (E12.0), clarifying the molecular dynamics of key cell types and lineages that develop into oral epithelial organs. The manuscript is extremely well written, and both the single-cell and imaging data are of very high quality. The study makes some important biological observations regarding the progressive transcriptional changes that specify different positional zones within the mandibular epithelium, and the signaling pathways likely to drive these changes. The findings of this study are likely to be of broad interest to both the craniofacial and broader developmental biology communities. Publication is recommended, provided that the authors address some points regarding data presentation and interpretation, as outlined below.

Comments for the author

Wnt and BMP signaling domains.
The authors make some striking observations that of broad expression gradients of Wnt and BMP signaling pathway components in the anterior mandibular epithelium (middle of page 10). The authors may wish to include a mention of the expression of Wnt and TGFB receptors and/or to employ one of the recent bioinformatic tools (e.g. CellChat, CellPhoneDB) to further explore whether sender/receiver relationships are likely to exist within the epithelium. Figure 2 indeed shows that Fzd10 is also expressed in the anterior epithelium, particularly in the V1/V2 zones. Do these potential sender/receiver relationships make any specific predictions about the effect of Wnt pathway perturbations on the progressive development of the A/D/P zones?

© 2022. Published by The Company of Biologists under the terms of the Creative Commons Attribution License (https://creativecommons.org/licenses/by/4.0/).

Progressive regionalization of the mandibular epithelium.
The RNAscope data showing the progressive specification of the A/D/P zones appears to be of high quality and is extremely compelling, particularly at the boundaries of these domains. The analysis of the timing and numbers of double-positive cells (Fig 7 panels M-O) suggests that individual cells at boundary regions can co-express markers of adjacent domains. Can the authors include some higher-resolution image examples of these double positive cells? An alternative hypothesis is that individual cells remain positive for only one domain, but intermingle for a brief period and later become physically sorted into adjacent domains. Higher resolution images of these cells (w/ channels separated) that clearly show co-expression of different zone markers in the same cell could help to better communicate that these trends are not simply due to cells overlapping in different z position of the image. It is also quite fascinating that the boundary between endoderm-derived (shh+) cells and zone P also appears to be similarly
"salt & pepper" at early stages, with an enormous amount of endoderm-derived shh signal E9.5-E11.5. Equally surprising was the result in Fig S8A that showed such a small number of endoderm/shh-derived cells in the mandibular epithelium at E12.
It could be interesting (though not essential for publication) to corroborate these lineage tracing results with another endoderm-specific CRE line, to better delineate the boundary between endoderm and ectoderm-derived cells.
Trajectories of zone states.
Given the dynamic trends observed for the zonal fates A/D/P, the authors propose a model in which cells with a dental-like identity at early timepoints shift to adopt a zone A fate at later timepoints. The Slingshot trajectory in Fig S8H is meant to provide supporting evidence of this fate switch. However, although the single cell/slingshot analyses indeed point to the existence of a continuum of states, the directionality of the state changes over time (D->A rather than A->D)
requires additional support. The authors may wish to attempt an RNA velocity analysis of their single-cell data, which might generate experimental support for D->A (vs A->D). An alternative hypothesis that could explain the shifting sizes of the A/D/P zone sizes (rather than direct fate conversion) is differential proliferation and/or cell death. The authors may wish to perform histological analysis of cell proliferation rates (e.g. BrdU/EdU, H3P, etc) and apoptosis rates
(e.g. caspase) to confirm that such rates are similar across zones and would not contribute significantly to changes in zone sizes.
[RNA velocity could also help validate some other state transitions predicted by the authors, e.g. basal->suprabasal->periderm within the P/S cluster]
Several aspects of the SCENIC analysis appearing in Fig 8 and Table S5 are confusing:
(1) The notation for representing regulatory networks in Fig 8 is difficult to interpret. Are the targets of the red genes only the gene names listed in black or all genes appearing below? Can the authors explain why some regulatory relationships are depicted and not others (e.g. Table S5 indicates that Sox2 and FoxP2 bind to both their own and each others' regulatory elements potentially suggesting a positive feedback loop).
(2) Where possible, can the nature of the regulatory interaction (positive vs repressive) be depicted in the figure?
(3) Figure 8 only displays a small subset of the targets for each TF. Is there statistical justification from the SCENIC analysis for displaying only these particular TF-target relationships? According to table S5 there are many additional targets, some of which might display expression patterns inconsistent with the well-behaved trends suggested by Fig 8. Can the authors include an assessment of the proportion of target TFs that display expected zonal patterns vs other trends?
(4) In the excel table for Table S5, the first worksheet is entitled "Table S3",
perhaps from an earlier version.
Other comments:
1. Single cell datasets, both raw reads, and genes x cells counts tables, should be made available in the appropriate online databases (e.g. NCBI-SRA and NCBI-GEO respectively)
2. Somewhere in the supplemental data, the authors should show a UMAP overlay with additional metadata flags for each cell (e.g. 6 biological replicates, gender) to confirm that these variables do not contribute major structures to the final UMAP atlas.
3. Some of the text elements with purple background in the figures are extremely hard to read
(e.g. the purple circle "Cluster 4" in Fig 1, and "IK" in Fig 4).

© 2022. Published by The Company of Biologists under the terms of the Creative Commons Attribution License (https://creativecommons.org/licenses/by/4.0/).

First revision

Author response to reviewers' comments

Reviewer 1 Advance Summary and Potential Significance to Field:
This manuscript describes scRNA-seq analysis of E12 mouse mandibular epithelia, with which the authors identified and verified the patterns of expression of several domain-specific markers and postulated potential gene regulatory networks patterning the mandibular epithelium along the aboral-oral axis. The authors propose an interesting hypothesis that the mandibular epithelium is initially dental-like, with the anterior and posterior regions subsequently specified to delineate the boundary of the tooth filed and confine the dental lamina to its position. They further proposed that the gradual sharpening of the dental lamina epithelium might be through mutual repression of region/cluster-specific transcription factors. Overall, the scRNA-seq dataset from the E12 mandibular epithelium will be a valuable resource and the hypothesized mechanism for regionalization of the mandibular epithelium is interesting. However, the manuscript is mostly descriptive and the major hypothesis remains to be tested.

Reviewer 1 Comments for the Author:
In this manuscript, the authors identified regionalization markers of the developing mouse embryonic mandibular epithelium at E12 using scRNA-seq and verified the patterns of expression of selected markers from E9.5 to E12 by in situ hybridization analysis. The bioinformatic analysis of the E12 mandible scRNA-seq dataset was thorough and well presented. The in situ validation data are of high quality. However, while the data generated several interesting hypotheses regarding mechanisms patterning the mandibular epithelium, the manuscript stops short of testing any of the major hypotheses. The only functional study included in the manuscript involved pharmacological inhibition of Ntrk2 in E11.5 mandibular explant cultures with which the authors showed reduction in dental epithelial proliferation in the incisor region but not the molar region at 48 hours after culture. However, several Ntrk2 mutant mouse lines have been reported and there has been no description of mandibular incisor defect, leaving the in vivo role of Ntrk2 in tooth development in question.
Thus, while the E12 mandible scRNA-seq dataset and the data analysis presented in Figures 1-8 provide a valuable resource to the developmental biology community, the lack of substantial functional studies to test some of the main hypotheses raised regarding the mechanisms regulating regionalization of the mandibular epithelium is a major limitation for this manuscript.

Response: We thank the reviewer for recognizing the quality of our scRNA-seq analysis and the effort to validate these results. While the data are largely descriptive, we believe that this is an important and comprehensive overview of the developing mandibular epithelium. Our hope is that this will be a valuable resource for the developmental biology community, while also generating interesting hypotheses for future investigations. For example, it will be important to examine the function of specific transcription factors in activating and/or inhibiting lineage-specific genes and how transcriptional boundaries are created. We plan to investigate these questions in our next study, utilizing mouse genetic mutants and embryonic stem cells expressing different transcription factors. Unfortunately, these experiments are outside the scope and the timeframe of the current study.

We did, however, added contents to the revised manuscript to address some of the concerns raised here. As Reviewer #3 pointed out, RNA velocity is a proven method for predicting cell lineages and is a reasonable test we could perform to begin answering whether anterior aboral cells and posterior oral cells are later patterned populations derived from progenitor cells expressing dental markers *Pitx2* and *Irx1*, as we proposed. We have therefore conducted a new round of scRNA-seq using cells from E9.5 mandibles and performed RNA velocity to infer lineages at the onset of oral-aboral patterning. Our data revealed that mandibular epithelial development in fact begins with a group of early populations that co-express *Vgll2*, *Pitx2*, and *Sox2*. Cells co-expressing these markers are already present when the mandibular arch is first formed at E8.5. Based on RNA velocity, these *Vgll2+/Pitx2+/Sox2+* cells first give rise to *Pitx2+/Irx1+* cells, which in turn produce *Tfap2b+* anterior aboral cells. We also performed RNA velocity on our E12.0 dataset, which showed that *Pitx2+/Irx1+* dental cells continue to give rise to anterior cells, but also posterior populations. Together these results support our original RNAscope analysis on the sequential formation of these populations and further refine the model of oral-aboral patterning.

© 2022. Published by The Company of Biologists under the terms of the Creative Commons Attribution License (https://creativecommons.org/licenses/by/4.0/).

We have incorporated the RNA velocity data, the expression of *Vgll2* and *Sox2* at E9.5-E11.0, and the updated model into Figure 7 (Fig. 7N-Q,W,X). Additional expression data of *Vgll2*, *Pitx2*, *Sox2*, and *Irx1* in the E8.5 arch ectoderm and feature plots of E9.5 epithelium were included in a new supplemental figure (Fig. S10G,H). We have also updated the corresponding sections in Results (under the heading "Mandibular epithelium undergoes progressive regionalization along the oral-aboral axis") and Discussion (under the heading "Patterning of the mandibular epithelium along the oral-aboral axis") to reflect these new results.

We also would like to thank the reviewer for pointing out the *in vivo* relevance of *Ntrk2* during tooth development. We are aware of the *Ntrk2* null mice previously reported, for example by Klein et al., 1993 and Rohrer et al., 1999. These papers described that most *Ntrk2* null mutants die shortly after birth and only some on non-C57BL6 background could survive (also via personal communication with Dr. Baoji Xu, who is the corresponding author on Rohrer et al). Dr. Xu's group has not examined *Ntrk2*$^{-/-}$ mutant teeth previously – unfortunately they no long maintain the line. Our plan is to generate *Crect; Ntrk2*$^{f/f}$ mice in the future to conditionally delete *Ntrk2* in the oral ectoderm and study its *in vivo* function during tooth development. We also would like to point out that tooth development is a robust process and early perturbation in cell proliferation doesn't always result in severe phenotypes subsequently. For example, both deletion of *Yap* (Liu et al., 2015) and deletion of *Smad7* (Liu et al., 2019) in the dental epithelium resulted in reduced epithelial proliferation, but tooth development was not abolished, and teeth are still formed with correct shapes, despite smaller in size.
These considerations are now included in the Discussion section, along with a reminder to readers that the *in vivo* role of *Ntrk2* requires further investigation. As the explant culture is a well-established experimental approach for studying tooth development, and our study here was carefully performed and quantified, we do believe that these results inform NTRK2's function and are worth being reported.

Klein, R., Smeyne, R. J., Wurst, W., Long, L. K., Auerbach, B. A., Joyner, A. L. and Barbacid, M. (1993). Targeted disruption of the trkB neurotrophin receptor gene results in nervous system lesions and neonatal death. Cell 75, 113–122.
Rohrer, B., Korenbrot, J. I., LaVail, M. M., Reichardt, L. F. and Xu, B. (1999). Role of neurotrophin receptor TrkB in the maturation of rod photoreceptors and establishment of synaptic transmission to the inner retina. J Neurosci 19, 8919–8930.
Liu, M., Zhao, S., Lin, Q. and Wang, X.-P. (2015). YAP regulates the expression of Hoxa1 and Hoxc13 in mouse and human oral and skin epithelial tissues. Mol Cell Biol 35, 1449–1461.
Liu, Z., Chen, T., Bai, D., Tian, W. and Chen, Y. (2019). Smad7 Regulates Dental Epithelial Proliferation during Tooth Development. J Dent Res 98, 1376–1385.

Reviewer 2 Advance Summary and Potential Significance to Field:
This resource paper describes the cell diversity of E12.0 mandibular epithelium using scRNA-seq and maps the identified cell populations using in situ hybridization. These studies have identified discrete populations, both in the developing ectodermal organs and in areas where the epithelium appears morphologically simple and uniform. Furthermore, they looked at the expression of a few oral-aboral marker genes at earlier development stages and hypothesize that mandibular ectoderm is initially more dental-like, but newly patterned anterior and posterior regions gradually expand and confine the dental progenitor field to its eventual position. Through computational analysis, this study identified key transcription factors and associated gene regulatory networks for different cell populations. For example, they tested the function of Ntrk2, a novel dental marker identified in this analysis, in the forming incisor using explant culture and discovered that NTRK2 promotes cell proliferation in the invaginating incisor placode. Altogether, this study provides a detailed atlas for the epithelial cell types in developing mandible, which serves as an important resource for generating new hypothesis and further investigation the function of specific genes/pathways during epithelial morphogenesis.

Response: We would like to first thank the reviewer for the positive feedback and for recognizing the value of this manuscript as an important resource for the community. The comments below are all very helpful and by addressing each of them, we believe that the manuscript is further improved.

© 2022. Published by The Company of Biologists under the terms of the Creative Commons Attribution License (https://creativecommons.org/licenses/by/4.0/).

Reviewer 2 Comments for the Author:
The scRNAseq work is generally well done and my concerns and comments are:

Major
1. Since ectoderm is only a very small portion in tissue sections, higher magnification images accompanied with high-quality nuclear staining images are highly recommended for the RNAscope results to see the ectodermal cell layers, such as Fig4B,C, Figure 7, and FigS5 C,D,G.

Response: This is a very important point and we have now enhanced DAPI signals in these images to better define the tissue outlines. We have also added additional magnified images to Figure 7 (C',D',H',I') and Figure S6 (D',E',G') (note that the original S5 is now S6) to show gene expression in individual cells of the ectodermal layer. Lastly, as the ectoderm is less distinct in younger embryos, we have performed additional experiments that combine RNAscope (for *Irx1*, *Pitx2*, and *Tfap2b*) with laminin immunostaining. Laminin labels the basement membrane in between the epithelium and the mesenchyme, thus allowing easier identification of RNAscope signals in the epithelium. These data complement the existing Figure 7 and are included in a new supplemental figure (Fig. S10A-D).

2. It is very helpful to have schematic drawings throughout the paper. It is recommended to incorporate the P/S and V2 clusters into the schematic drawing if possible.

Response: We have now added clusters P/S and V2 to our schematics – thank you for the suggestion.

3. It is interesting that the suprabasal cells are clustered with the periderm. It is not clear what the P/S1 and P/S3 cells are since the marker genes used for these clusters are expressed in the corresponding basal cells. To address this, it is recommended to double label with a marker which is expressed only in P/S1 and P/S3 but not in the corresponding basal cells.

Response: We similarly have wanted to identify unique markers for these subclusters (P/S1 and P/S3) to be able to more cleanly label them on sectioned tissues. Unfortunately, the best markers are ones associated with anterior or posterior regional identities, and thus expressed in both the basal layer and the periderm (note that in the anterior and posterior epithelium, most suprabasal cells are simply peridermal cells, and there are few suprabasal cells in between the two layers). To circumvent this, we utilized the pan-P/S marker *Zfp750* to label all suprabasal and peridermal cells (added as Fig. S6C) and used that as a guide to distinguish P/S1 and P/S3 populations that respectively express *Tfap2b*/*Cxcl14* (P/S1) and *Dmrt2* (P/S3). We now include these results, along with enlarged images showing co-expression of *Zfp750* with *Tfap2b*, *Cxcl14* or *Dmrt2*, in Supplemental Figure S6 D,E,G. Finally, to clarify that P/S1-3 contain both suprabasal and peridermal cells, and that P/S4 contains only peridermal cells of a particular subtype, we have added the following modified sentence in the corresponding "Suprabasal populations" section in Results: "P/S1-3 therefore comprise peridermal and suprabasal cells that retain positional identities. P/S4 contains a peridermal subtype population that disperses over the epithelial surface and expresses novel markers *Tagln* and *Acta2*." We hope these changes better describe the different P/S subclusters.

4. What is the Seurat package version? Which R version was used? For Metascape, were any p-value cutoffs used for the function enrichment? Were any other filters used for the enriched terms? What types of GO terms were shown in the results? Were merged or overlapped marker genes used for the analysis of multiple clusters?

Response: We thank the reviewer for pointing out these important experimental details that were omitted previously. Seurat version 4.0 and R version 4.1.2. were used and we have included this information in Methods now.

For Metascape analysis, we used the default cutoff P-value of 10^{-2} to identify enriched gene functions. For GO terms that are not sufficiently informative (e.g. terms like "tissue morphogenesis" or "response to growth factors"), or not directly relevant to epithelial development (e.g. "mesenchyme development", "muscle tissue development", or "ossification"), or duplicated (e.g. "cell-cell signaling by wnt" overlaps with "Wnt signaling pathway"), we have excluded them from our figures. GO terms shown in the figures are therefore curated lists that

© 2022. Published by The Company of Biologists under the terms of the Creative Commons Attribution License (https://creativecommons.org/licenses/by/4.0/).

help us understand how these enriched genes could contribute to the development and functions of different epithelial populations. To clarify how these results were generated, we have included the following sentence in the Methods section, "…the default cutoff P-value 10^{-2} was used. Top ranked GO terms that better inform signaling pathways or cell biological functions and processes in the context of epithelial development are selected for presentation in figures." To make the rest of the GO terms accessible to readers, we have also included a new supplemental Table (Table S3) in the revised manuscript.

For Metascape analysis of multiple clusters, their markers were merged, and duplicated genes were removed before the analysis. We now clarify this in the methods section with the following sentence, "For clusters with similar expression profiles and gene functions, their marker genes were merged and collapsed as a single input for the Metascape analysis."

5. Ntrk2 inhibitor treatment causes smaller tooth buds than controls. However, more proliferation was observed in the treated incisor IK region. Was this because the IK was not formed in the treated tissue?

Response: We have too speculated that the IK was not properly formed in samples treated with the NTRK2 inhibitor. To test this, we have repeated the experiment and processed the samples for *Shh* RNAscope staining followed by p21 immunostaining. *Shh* is one the earliest markers for the IK (beginning at E11.0, see Fig. 7I) and p21 is a cell cycle inhibitor that accompanies IK differentiation to induce cell cycle arrest in the IK (Du et al., 2017; Ahtiainen et al., 2016). Interestingly, while *Shh* was expressed in the treated samples, p21 was completely gone. As we were using E11.5 embryos in our explant culture experiment, it is perhaps not surprising that *Shh* expression remained. However, inhibition of NTRK2 signaling in the epithelium and/or mesenchyme could indirectly disrupt full IK differentiation, and in the absence of p21, proliferation was maintained in the ANA-12-treated IK. These results are now included in Supplemental Figure S14 to help explain the EdU results in the IK in Fig. 9C,D.

Ahtiainen, L., Uski, I., Thesleff, I. and Mikkola, M. L. (2016). Early epithelial signaling center governs tooth budding morphogenesis. *J. Cell Biol.* 214, 753-767.
Du, W., Hu, J. K., Du, W. and Klein, O. D. (2017). Lineage tracing of epithelial cells in developing teeth reveals two strategies for building signaling centers. *J. Biol. Chem.*

6. A data availability section should be included. It is strongly recommended to deposit not only the raw data but also the analyzed scRNA-seq data, including the R objects, in a public repository.

Response: We have now uploaded the raw data, the barcode.tsv, genes.tsv and matrix.mtx files from 10x, as well as the Seurat objects to GEO. The accession number (GSE202921) is provided under the data availability section.

7. For test bud markers were detected in E12.5 tissues in Fig6 E and FigS7B-E and were not detected at E12.0 samples in FigS7B-E by in situ. However, they were detected in the E12.0 scRNA-seq. This discrepancy should be addressed.

Response: These markers are expressed at a lower level in the tongue at E12.0 than at E12.5, and while scRNA-seq could detect their expression, whole mount *in situ* hybridization may not be sensitive enough. As RNAscope in general is a more sensitive detection method, we re-examined the expression of *Shh*, *Dsc3*, and *Prss23* in the E12.0 tongue. These results demonstrated that these markers are indeed expressed in the tongue at E12.0, although more diffused than at E12.5 (which could also contribute to the more prominent punctate staining seen in E12.5 whole mount samples). These new images are added to Supplemental Figure S8B-D now. Finally, we have also enhanced the original E12.0 whole mount staining to better show the low- level staining in the tongue at that timepoint.

Minor

1. The last line on the first page, "The mouse jaw" should be "The mouse lower jaw".

Response: Thanks for catching this and we have now corrected it.

© 2022. Published by The Company of Biologists under the terms of the Creative Commons Attribution License (https://creativecommons.org/licenses/by/4.0/).

2. In the Methods, on page 4, at the end of paragraph 2, “We have also regressed out genes encoding lincRNAs, Gm42418 and AY036118….”. It is more appropriate to say “removed” instead of “regressed out” if this was done in the same way as in the reference.

Response: We have indeed regressed out these two genes using AddModuleScore and the reference was originally cited to provide the rationale of doing such a regression, as we did find that these two genes contributed to the clustering of a very small population initially. To avoid any confusion, we have added “Using AddModuleScore,” in front of the sentence, “we have also regressed out genes encoding lincRNAs, Gm42418 and AY036118…” in the Methods section. Hopefully this made it clear.

3. What does the “mirrored clusters” in first paragraph of the Results section mean?

Response: Among all the clusters identified in the beginning, many are paired; one expresses cell cycle genes, while the other does not. They otherwise have very similar expression profiles. We described them as “mirrored” because they occupy the opposite sides of the UMAP. This, however, is likely confusing as there’s no real symmetry there. We have modified the sentence as “…contains several matching clusters segregated by the expression of cell cycle genes…”. We have also added in the Figure S1 legend the following sentence, “Differences in cell cycles contribute greatly to the initial clustering, separating out clusters with otherwise closely matched expression profiles into cycling (bottom half) and non-cycling (top half) populations”.

4. Figure 2C the label on the left side is off one gene for the dental lamina and P/S. Scube1 is a not a marker for P/S, but for dental lamina.

Response: Thank you for spotting this! We have moved the brackets to the correct position to include *Scube1*
in the dental lamina list.

5. The table names do not match the table names for Table S3-5.

Response: The supplemental table names are now corrected. Note that we have included a new supplemental table (Table S3) that contains all the GO terms.

Reviewer 3 Advance Summary and Potential Significance to Field:
The manuscript by Ye et al succeeds in delivering a comprehensive single-cell expression atlas of the developing mandibular epithelium (E12.0), clarifying the molecular dynamics of key cell types and lineages that develop into oral epithelial organs. The manuscript is extremely well written, and both the single-cell and imaging data are of very high quality. The study makes some important biological observations regarding the progressive transcriptional changes that specify different positional zones within the mandibular epithelium, and the signaling pathways likely to drive these changes. The findings of this study are likely to be of broad interest to both the craniofacial and broader developmental biology communities. Publication is recommended, provided that the authors address some points regarding data presentation and interpretation, as outlined below.

Response: We appreciate the positive response and the constructive feedback from the reviewer. As you will see below, we have performed additional analyses, including CellChat, RNA velocity, and expression studies, as suggested by the reviewer. We believe that these results help improve the manuscript and provide further insights into how different mandibular epithelial populations may arise.

Reviewer 3 Comments for the Author:
Wnt and BMP signaling domains.
The authors make some striking observations that of broad expression gradients of Wnt and BMP signaling pathway components in the anterior mandibular epithelium (middle of page 10). The authors may wish to include a mention of the expression of Wnt and TGFB receptors and/or to employ one of the recent bioinformatic tools (e.g. CellChat, CellPhoneDB) to further explore whether sender/receiver relationships are likely to exist within the epithelium. Figure 2 indeed

© 2022. Published by The Company of Biologists under the terms of the Creative Commons Attribution License (https://creativecommons.org/licenses/by/4.0/).

shows that Fzd10 is also expressed in the anterior epithelium, particularly in the V1/V2 zones. Do these potential sender/receiver relationships make any specific predictions about the effect of Wnt pathway perturbations on the progressive development of the A/D/P zones?

Response: We agree with the reviewer that it will be both interesting and important to further explore our observation of enriched WNT and BMP signaling molecules in the anterior epithelium. To that end, we applied CellChat to our E12.0 dataset. The results support the initial gene ontology analysis and indicate that the anterior epithelium can function as a "WNT sender" and a "BMP influencer". In addition, as the reviewer pointed out, these signaling processes could indeed take place within the anterior epithelium, based on the detection of WNT/BMP ligand-receptor pairs in these clusters. The sender/receiver relationships are also captured using the "Chord diagram" function of the package. These new data are now presented as Figure 3I- L, as well as in a new supplemental figure (Fig. S5). In the same supplemental figure, we have also included the incoming and outgoing river plots and heatmaps for all the epithelial clusters. These graphs show key signaling events in other populations, such as the production of BMP/FGF/HH signals from the initiation knot and the involvement of NOTCH signaling in the peridermal cells; both cited in their respective result sections. Finally, although both our data here and a recently published paper by Van Otterloo et al., 2022 suggest that there is high WNT signaling in the anterior epithelium, it is perhaps difficult to predict how WNT pathway perturbation would affect oral-aboral patterning, especially when the responsiveness to WNT is graded and likely modulated by other factors. We therefore only postulated how differential WNT responses can be achieved in the epithelium in the Discussion section, without making major predictions on potential experimental outcomes. This is, however, a very interesting question that we would like to investigate in the future.

Van Otterloo, E., Milanda, I., Pike, H., Thompson, J. A., Li, H., Jones, K. L. and Williams, T. (2022). AP-2α and AP-2β cooperatively function in the craniofacial surface ectoderm to regulate chromatin and gene expression dynamics during facial development. *eLife* 11, e70511.

Progressive regionalization of the mandibular epithelium.
The RNAscope data showing the progressive specification of the A/D/P zones appears to be of high quality and is extremely compelling, particularly at the boundaries of these domains. The analysis of the timing and numbers of double-positive cells (Fig 7 panels M-O) suggests that individual cells at boundary regions can co- express markers of adjacent domains. Can the authors include some higher-resolution image examples of these double positive cells? An alternative hypothesis is that individual cells remain positive for only one domain, but intermingle for a brief period and later become physically sorted into adjacent domains. Higher resolution images of these cells (w/ channels separated) that clearly show co-expression of different zone markers in the same cell could help to better communicate that these trends are not simply due to cells overlapping in different z position of the image.

Response: This is an excellent point, and we should have included images that better show co-expression of these markers in the same cell. High magnification images focusing on regions with overlapping signals are now included in Figure 7 (B'-D', G'-I'). These results show that cells do co-express markers from neighboring regions and likely switch to a specific regional program later as the boundary sharpens, thus similar to hindbrain segmentation. This would also agree with our own observation from live imaging that there is no obvious cell sorting behavior within the basal layer (data not included in this manuscript). We hope to in the future generate mouse lines that express fluorescent proteins from key markers' loci and perform live imaging to confirm whether cell sorting accompanies transcriptional switching.

It is also quite fascinating that the boundary between endoderm-derived (shh+) cells and zone P also appears to be similarly "salt & pepper" at early stages, with an enormous amount of endoderm-derived shh signal E9.5- E11.5. Equally surprising was the result in Fig S8A that showed such a small number of endoderm/shh-derived cells in the mandibular epithelium at E12. It could be interesting (though not essential for publication) to corroborate these lineage tracing results with another endoderm-specific CRE line, to better delineate the boundary between endoderm and ectoderm-derived cells.

© 2022. Published by The Company of Biologists under the terms of the Creative Commons Attribution License (https://creativecommons.org/licenses/by/4.0/).

Response: We were also puzzled by the lack of *Shh*^{CreER}-labelled endodermal descendants in the tongue, given what we observed using *Shh* RNAscope. Two factors likely contributed to this: 1) RNAscope is very sensitive and likely picked up weak expression that didn't translate into CreER-based cell labelling; 2) we gave tamoxifen at a lower dose than usual at E8.5, as we were avoiding labelling of IK cells at E11.0 by residual tamoxifen in the system, as well as tamoxifen toxicity to young embryos. While we do not have time to acquire another endoderm-specific Cre line, we have optimized our dosing scheme for inducing Shh-CreER activation (a higher dosage of tamoxifen at E9.5). This resulted in labelling of more cells in the posterior tongue (a new image now shown as Fig. S9A), thus better matching our description in the texts, as well as previous findings using the *Sox17-2A-iCre* mouse line.

Trajectories of zone states.
Given the dynamic trends observed for the zonal fates A/D/P, the authors propose a model in which cells with a dental-like identity at early timepoints shift to adopt a zone A fate at later timepoints. The Slingshot trajectory in Fig S8H is meant to provide supporting evidence of this fate switch. However, although the single cell/slingshot analyses indeed point to the existence of a continuum of states, the directionality of the state changes over time (D->A rather than A->D) requires additional support. The authors may wish to attempt an RNA velocity analysis of their single-cell data, which might generate experimental support for D->A (vs A->D). An alternative hypothesis that could explain the shifting sizes of the A/D/P zone sizes (rather than direct fate conversion) is differential proliferation and/or cell death. The authors may wish to perform histological analysis of cell proliferation rates (e.g. BrdU/EdU, H3P, etc.) and apoptosis rates (e.g. caspase) to confirm that such rates are similar across zones and would not contribute significantly to changes in zone sizes. [RNA velocity could also help validate some other state transitions predicted by the authors, e.g. basal->suprabasal->periderm within the P/S cluster]

Response: The suggestion of conducting RNA velocity is an excellent idea and we have now included it in our study. However, as the E12.0 data are temporally more removed from when aboral cells are first established around E9.5, we have conducted a new scRNA-seq experiment using mandibular cells from that stage to specifically examine the lineage directionality between the oral and aboral cells. Please refer to our response to Reviewer #1's comments for a more detailed description of our findings. But briefly, E9.5 RNA velocity showed that zone A founder cells are derived from zone D cells, which themselves were progeny of a group of *Vgll2+/Pitx2+/Sox2+* ectodermal progenitor cells. These results would also match the spatiotemporal expression patterns of different oral and aboral markers. To see if cells might behave similarly at a later stage, we also conducted RNA velocity using our E12.0 dataset. This showed that zone D cells continue to give rise to neighboring zone A cells, but also posterior zone P cells. Together, these results support our original RNAscope analysis of regional markers and help refine our model of oral-aboral patterning. (We however did not propose that suprabasal cells give rise to the periderm. As mentioned in the introduction, the periderm is formed very early on and other suprabasal cells are formed later from the basal layer, especially in the placodes. Consistent with this, we could see velocity vectors flow from clusters representing the zone D basal layer to the periderm cluster at E9.5 and the P/S cluster at E12.0. However, performing RNA velocity using only the E12.0 periderm cells was not informative as arrows were very short and the directionality was not obvious, likely due to the fact that these cells are formed from the basal layer and not from a specific P/S subcluster. We hope that the reviewer agrees that we don't present this particular analysis, as we cannot draw too much conclusion from it.)

The reviewer also raised a good point on whether there are differential proliferation/apoptosis rates in different regions. As apoptosis is rarely seen in these early stages (our own observations and Fujita et al., 2013; MacKenzie et al., 2009), we focused on cell proliferation. We combined EdU labelling (EdU pulse/chase for 30 minutes) with *Tfap2b/Pitx2* RNAscope in E10.5 embryos to determine if proliferation rates differ between these regions as they shift in their relative sizes. While proliferation is mostly uniform throughout the oral ectodermal surface, proliferation is reduced in the more ventral/aboral epithelium, suggesting that these cells are more differentiated and becoming post-mitotic. As a result, we don't think differential proliferation between zones is the main driver for narrowing the dental field. These results are now included in Supplemental Figure S10E,F. We plan to in the future study how

© 2022. Published by The Company of Biologists under the terms of the Creative Commons Attribution License (https://creativecommons.org/licenses/by/4.0/).

switching in the zonal transcriptional programs could lead to the refinement of the dental field.

Fujita, K., Taya, Y., Shimazu, Y., Aoba, T. and Soeno, Y. (2013). Molecular signaling at the fusion stage of the mouse mandibular arch: involvement of insulin-like growth factor family. Int J Dev Biol 57, 399–406.
MacKenzie, B., Wolff, R., Lowe, N., Billington, C. J., Peterson, A., Schmidt, B., Graf, D., Mina, M., Gopalakrishnan, R. and Petryk, A. (2009). Twisted gastrulation limits apoptosis in the distal region of the mandibular arch in mice. Developmental Biology 328, 13–23.

Several aspects of the **SCENIC analysis** appearing in Fig 8 and Table S5 are confusing:
(1) The notation for representing regulatory networks in Fig 8 is difficult to interpret. Are the targets of the red genes only the gene names listed in black, or all genes appearing below? Can the authors explain why some regulatory relationships are depicted and not others (e.g. Table S5 indicates that Sox2 and FoxP2 bind to both their own and each others' regulatory elements potentially suggesting a positive feedback loop).

Response: We apologize for the less-than-ideal figure presentation. Our initial intention for Fig. 8A was to demonstrate how these key transcription factors (TFs) may target the different cluster markers that are either used in the manuscript for mapping or reflecting the key function of the epithelial region. A few arrows were also used to depict some of the additional transcriptional interactions discussed in the texts. We were concerned that plotting all the predicted bindings will make this a very complex figure. However, we agree with the reviewer that omitting these transcriptional interactions creates confusion. As a result, we have updated the figure to capture interactions that were excluded initially. Some of the main changes we made: 1) we used blue boxes for TFs and gray boxes for targets, and added an arrow from each TF to its targets immediately below; 2) we added a circled arrow to indicate TF binding to its own promoter; 3) additional TF-target associations were depicted using black arrows. While this is busy, we believe that this is a good balance between readability and being too complex (e.g. using different colors or line thickness to separate out different TFs).

(2) Where possible, can the nature of the regulatory interaction (positive vs repressive) be depicted in the figure?

Response: Unfortunately, SCENIC does not provide direct repressive interactions based on negative- correlations in the TF modules (co-expression of TFs and targets) due to low motif enrichment (Aibar et al., 2017). While most of the TFs identified from SCENIC in this study are transcription activators, several of them can also function as repressors. However, their role as an activator (to promote the expression of co- expressed targets) or a repressor (to dampen/modulate the expression of co-expressed targets) within the co- expression module cannot be immediately determined. For instance, while we discussed TRPS1 as a potential repressor for GATA3 targets, TRPS1 can also function as an activator at GATA3 (and other) binding sites (Fantauzzo and Christiano, 2012; Witwicki et al., 2018). To avoid confusion on this matter, we have updated our texts to clarify that the promotive and repressive functions of TRPS1 are context dependent. Finally, because of these reasons, we simply use arrows to indicate putative TF-target bindings without defining positive vs repressive outcomes.

Aibar, S., González-Blas, C. B., Moerman, T., Huynh-Thu, V. A., Imrichova, H., Hulselmans, G., Rambow, F., Marine, J.-C., Geurts, P., Aerts, J., et al. (2017). SCENIC: single-cell regulatory network inference and clustering. Nat Methods 14, 1083-1086.
Fantauzzo, K. A. and Christiano, A. M. (2012). Trps1 activates a network of secreted Wnt inhibitors and transcription factors crucial to vibrissa follicle morphogenesis. Development 139, 203–214
Witwicki, R. M., Ekram, M. B., Qiu, X., Janiszewska, M., Shu, S., Kwon, M., Trinh, A., Frias, E., Ramadan, N., Hoffman, G., et al. (2018). TRPS1 Is a Lineage-Specific Transcriptional Dependency in Breast Cancer. Cell Rep 25, 1255-1267.e5.

(3) Figure 8 only displays a small subset of the targets for each TF. Is there statistical justification from the SCENIC analysis for displaying only these particular TF-target relationships? According to table S5 there are many additional targets, some of which might display expression patterns inconsistent with the well-behaved trends suggested by Fig 8. Can the authors include

© 2022. Published by The Company of Biologists under the terms of the Creative Commons Attribution License (https://creativecommons.org/licenses/by/4.0/).

an assessment of the proportion of target TFs that display expected zonal patterns vs other trends?

Response: As mentioned in (1) above, the list of curated genes in Figure 8 were selected either because they were used in this study to map the corresponding clusters or because they reflect the main function of the region (e.g. WNTs in zone A or desmosomes/tight junctions in P/S). To clarify that this is a curated list, we have added the following in the text: “Fig. 8 shows curated interactions; full lists in Table S6”. (note that the original Table S5 is now S6)

To assess if other targets would follow the same zonal patterns, one approach is to examine the expression correlation between these targets and their TFs. As it will not be possible to show individual targets in a figure form, we generated feature plots of individual TFs and their targets’ module scores (created using Seurat, showing the average expression of a gene list) in a new supplemental figure (Fig. S11). These results show qualitatively that TFs and their targets have similar zonal expression patterns. This is expected as the targets were identified based on their co-expression with TFs. Another way to demonstrate this is to assess the Spearman correlation between individual targets and their predicted TFs. Randomly chosen genes would likely have no correlation; while predicted targets exhibit positive correlations. To generate the Spearman correlation of random genes, we calculated the correlation of all the genes with each TF, then randomly picked 1500 genes for plotting. (We chose 1500 genes because that is close to the number of targets predicted by SCENIC.) Plotting the Spearman correlation of “actual predicted targets with their TF” vs “randomly selected targets with each TF” showed that predicted targets have a positive correlation; while random genes center around 0 correlation. Image below shows the distribution of correlations for Regulon predicted targets (blue), and correlations between random genes with Gata3 (purple), Grhl3 (red), Irx1 (green), and Foxc1 (yellow).

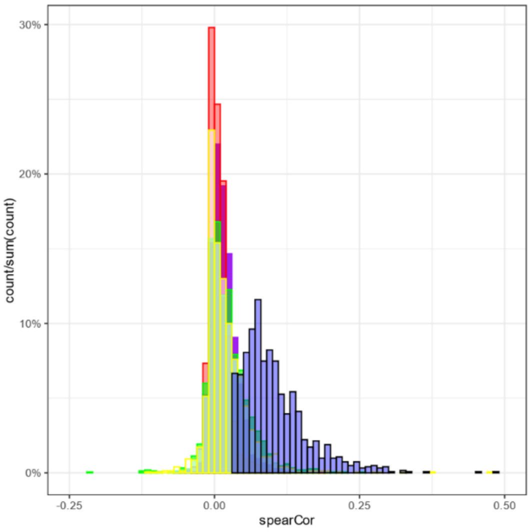

The same results were obtained when correlations of random genes were generated using TF module scores from each epithelial region (i.e. correlation with the average expression of zonal TFs). These results are presented instead of individual TFs in Fig. S11 in order to fit within the figure space. Together, we hope these provide a measure of how the expression of other predicted targets would similarly follow the zonal patterns depicted in Figure 8A.

(4) In the excel table for Table S5, the first worksheet is entitled “Table S3”, perhaps from an earlier version.

Response: Thank you for spotting this. We have now corrected this error, but changed it to Table S6, as a new Supplemental Table was added to make the Metascape gene ontology results accessible.

Other comments:

1. Single cell datasets, both raw reads, and genes x cells counts tables, should be made available in the appropriate online databases (e.g. NCBI-SRA and NCBI-GEO, respectively)

© 2022. Published by The Company of Biologists under the terms of the Creative Commons Attribution License (https://creativecommons.org/licenses/by/4.0/).

Response: We have now uploaded the raw reads (fastq), the barcode.tsv, genes.tsv and matrix.mtx files from 10x, as well as the Seurat objects to SRA and GEO. The accession number (GSE202921) is provided under the data availability section.

2. Somewhere in the supplemental data, the authors should show a UMAP overlay with additional metadata flags for each cell (e.g. 6 biological replicates, gender) to confirm that these variables do not contribute major structures to the final UMAP atlas.

Response: The 6 biological samples were pooled together prior to single cell dissociation and partitioning in order to achieve sufficient cell numbers. It is therefore not 6 biological replicates. To prevent any confusion, we have gone through our Methods section to make sure that this is clear. Due to COVID and costs, we only performed 1 sequencing experiment. However, we have relied on extensive *in situ* mapping to verify the scRNA-seq results. In terms of differential clustering due to gender differences, the initial UMAP did segregate clusters based on gender related genes (e.g. *Xist*, *Ddx3y*, *Uty*, etc.), especially after regressing out cell cycle genes. We have therefore regressed out gender related genes in our analysis as well. Relevant feature plots for representative gender related genes are now included in Fig. S1.

3. Some of the text elements with purple background in the figures are extremely hard to read (e.g. the purple circle "Cluster 4" in Fig 1, and "IK" in Fig 4).

Response: We have now changed these texts to white, thus improving its contrast to the purple background.

Second decision letter

MS ID#: DEVELOP/2022/200539

MS TITLE: Understanding the development of oral epithelial organs through single cell transcriptomic analysis

AUTHORS: Qianlin Ye, Arshia Bhojwani, and Jimmy K. Hu
ARTICLE TYPE: Research Article

I am happy to tell you that your manuscript has been accepted for publication in Development, pending our standard ethics checks.

Reviewer 1

Advance summary and potential significance to field

The revised manuscript is improved.

Comments for the author

No additional comment.

Reviewer 2

Advance summary and potential significance to field

The authors have addressed all my concerns adequately. I believe that this resource manuscript is suitable for publication in Development.

© 2022. Published by The Company of Biologists under the terms of the Creative Commons Attribution License (https://creativecommons.org/licenses/by/4.0/).

Comments for the author

No further revisions required.

Reviewer 3

Advance summary and potential significance to field

The manuscript by Ye et al succeeds in delivering a comprehensive single-cell expression atlas of the developing mandibular epithelium (E12.0), clarifying the molecular dynamics of key cell types and lineages that develop into oral epithelial organs. The manuscript is extremely well written, and both the single-cell and imaging data are of very high quality. The study makes some important biological observations regarding the progressive transcriptional changes that specify different positional zones within the mandibular epithelium, and the signaling pathways likely to drive these changes. The findings of this study are likely to be of broad interest to both the craniofacial and broader developmental biology communities.

Following the initial round of review, the authors have made considerable and significant improvements to the manuscript. This reviewer particularly appreciates the addition of high-resolution images confirming gene expression colocalization and RNA-velocity analyses (Fig 7), as well as the clarifications to the Scenic analyses and EdU experiments. Together these additions have greatly strengthened the principal claims of the manuscript. Publication is highly recommended.

Comments for the author

Following the initial round of review, the authors have made considerable and significant improvements to the manuscript. This reviewer particularly appreciates the addition of high-resolution images confirming gene expression colocalization and RNA-velocity analyses (Fig 7), as well as the clarifications to the Scenic analyses and EdU experiments. Together these additions have greatly strengthened the principal claims of the manuscript. Publication is highly recommended.

© 2022. Published by The Company of Biologists under the terms of the Creative Commons Attribution License (https://creativecommons.org/licenses/by/4.0/).
